# A defensive model and implementation baseline for the metaverse and extended reality systems

**DOI:** 10.7717/peerj-cs.3054

**Published:** 2025-08-29

**Authors:** Sara Qamar, Hasan Tahir, Zahid Anwar, Naveed Ahmed, Shahzaib Tahir, Muhammad Aleem

**Affiliations:** 1 Department of Information Security, National University of Sciences and Technology (NUST), Islamabad, Pakistan; 2 North Dakota State University, Fargo, ND, USA; 3 School of Science, Coventry University, England, United Kingdom

**Keywords:** Metaverse, Extended reality (XR), Proactive defense, Security and privacy, Interoperability, Standards and regulations, Human-Computer interaction, Implementation SDKs, Authenticity

## Abstract

The metaverse and extended reality (XR) systems are vulnerable to emerging security threats, as developers have prioritized competitive business gains over security. The virtual entities, immersive experiences, and lack of centralized governance pose significant challenges in establishing standardized guidelines for XR systems and its stakeholders. In this research, a panoramic view is presented to identify mitigation strategies and defensive capabilities, including authenticity, privacy, integrity, interoperability, virtual forensics, and incident reporting to counter potential threats. To facilitate the implementation of a secure XR system, a novel baseline model is introduced, outlining key attributes and functions aligned with the available libraries. A statistical analysis is performed to assess the quality and effectiveness of development resources in embedding novel XR security features. Furthermore, this research assesses the security posture of prominent XR systems and examines the applicable regulatory frameworks in immersive environment. Finally, security recommendations are proposed to counter the threat landscape of XR and the metaverse.

## Introduction

Extended reality (XR) is an umbrella term that refers to all immersive technologies comprising virtual reality (VR), augmented reality (AR), and mixed reality (MR). XR is also known as cross-reality, which represents the overlapping AR, VR, and MR experiences. It is a blend of physical and digital world thus producing an immersive environment. The XR technologies are widely adopted in diverse spheres of life including healthcare, sports, games, social media, medical, military, business, TV & films, retailers, and training (Yildirim, 2024). The generation and rendering of XR overlays and virtual objects in the physical world require the user’s personal, physical, environmental, and real life input, which involves gaze monitoring, body tracking, sensory gadgets attached to the human body, continuous behavioral monitoring, and locomotion data. The immersive experiences in XR are generated by integrating several evolving technologies comprising spatial computing, 5G, blockchain, virtual currency, non-fungible tokens (NFTs), BCI (brain-computer interface) technologies, head-mounted displays, and body and motion trackers. The evolving metaverse is considered as a ’master of information technology’, and is still at its preliminary phase of development (Guangjun et al., 2023). The metaverse potential and its impact on society, human psychology, culture, and governance is yet to be fully explored.

The implementation of security controls in these memory-constrained and sophisticated hardware devices is very complex. The blend of these technologies has amplified the security vulnerabilities and potential threats, raising security and privacy concerns (Noah, Shearer & Das, 2022). The novel sophisticated attacks applicable to the immersive environment include but are not limited to virtual harassment, deepfakes, avatar impersonation attacks, virtual currency scams, motion sickness, avatar teleportation attacks, virtual data inference, bystander privacy violations, emotion hacking, theft of virtual identities, disorientation attacks (Huang, Li & Cai, 2023; Kang, Koo & Kim, 2023).

It has been observed that the XR enterprises are more likely to be target of cyber attacks, as compared to other businesses, the increase in bot attacks was 80%, and human-launched cyber attacks were 40% (Arkose Labs, 2022). The XR ecosystem lacks a centralized governing entity, the stakeholders operate with varying incentives, diverse regional backgrounds, and legal liabilities. The lack of globally accepted standards, regulations, and privacy compliance certificates further exacerbates interoperability concerns. The XR systems pose legal accountability challenges in ensuring the security and privacy of consumers.

Security vulnerabilities often arise when developers overlook security considerations during implementation, sidestepping established best practices and security standards. It is imperative to integrate robust security measures during the initial stages of development for preemptive mitigation of vulnerabilities and loopholes within the integrated XR systems. To fortify XR systems against potential threats, it is essential to incorporate advanced security features proactively. Robust security features would enhance users’ trust in acquiring metaverse services and will safeguard users against immersive attacks.

Existing research in defensive approaches to counter metaverse and XR threats are limited because of the evolution of technology and the emerging need for new security mechanisms to counter the novel XR threats, immersive vulnerabilities, and potential virtual crimes. The available research addresses only a small subset of the security challenges faced in the entire XR and the metaverse ecosystem or focuses on a specific security approach. We did not encounter any research presenting a panoramic view of defensive mechanisms to ensure authenticity, privacy, integrity, interoperability, virtual forensics, and incident reporting with the supportive implementation attributes for the metaverse and XR systems. The related research work is summarized below.

### Related work

The metaverse brings new devices and sensors that offer a variety of biometric verification and sensing techniques for identity management. Jarin et al. (2023) proposes a model named “*BehaVR*,” aimed at VR user identification and investigates the capabilities of potential adversaries who can access the VR sensors data from VR apps and VR devices using installed APIs. The authors employ this framework to conduct a user study on the Quest Pro platform and examine real user interaction in VR. Explores the methodology through which user identities can be identified within applications on the device that demonstrate analogous user behaviors. Research by Lin & Latoschik (2022) raises concerns regarding potential threats to avatars’ identity and privacy in social VR applications and discusses the available solutions to protect the representation of digital bodies or personalized realistic avatars.

Zhang et al. (2017) presented an authentication scheme (AugAuth) that utilizes augmented reality (AR) displays and commercial off-the-shelf (COTS) gesture control sensors as input devices. The scheme provides a user input interface visible exclusively to the user and generates a unique interface for each authentication attempt. They tackle challenges in electromyogram signal processing, including signal annotation and finger classification to enable user input through finger movements. Wang et al. (2023) proposed a multi-attribute authentication approach for VR experience that involves rendering various 3D objects with distinct attributes. VR users select combinations of 3D objects and their associated attributes for authentication. The authors performed three user studies on usability, security, and memorability. The results prove that the approach mitigates shoulder surfing and MITR (man in the room) attacks. The researchers explore metaverse authentication using unique biometric features like electroencephalography (EEG) (Anastasaki et al., 2023), iris (Wang, Li & Yan, 2023), *etc*. Stephenson et al. (2022) compare the existing AR/VR authentication schemes with the strategies adopted by developers and embedded in devices. The author’s research identified user-friendly schemes and highlighted the authentication attributes of AR/VR devices. Cheng, Chen & Han (2023) aimed at achieving zero-trust user authentication for virtual reality (VR), investigating biometrics-based authentication techniques for continuous verification of VR users by incorporating multi-modal data. The research utilizes federated learning (FL) to safeguard user privacy of biometric data. Initial investigations reveal that conventional FL algorithms do not effectively support biometric-based authentication for VR users, resulting in accuracy levels below 10%. The article explores the underlying reasons for this issue and outlines the associated challenges. George et al. (2017) research has investigated the adoption and evaluation of existing authentication schemes in virtual reality based on PIN and pattern unlock. Bozkir et al. (2023) highlights the importance of eye-tracking in VR settings, and how the combination of eye-tracking data with stimulus-related information can reveal privacy-sensitive attributes. The authors also elaborate on the eye-based authentication schemes.

Noah, Shearer & Das (2022) identified the number of vulnerabilities (Common Vulnerabilities and Exposures (CVE) count) and the implemented security controls in 10 AR/VR devices, including Google Glass, HTC Vive, Oculus Quest, and others. The authors manually scanned the web page content of XR devices to determine the existing vulnerabilities, with the available security controls for authentication and access control mechanisms like AES-256, RSA, *etc*. The Ellysse Dick report (Dick, 2021) highlights that AR/VR devices operate on users’ data which aggravates the consumer’s privacy concerns. The established regulatory frameworks, such as the Health Information Portability and Accountability Act (HIPAA) and the Children’s Online Privacy Protection Act (COPPA), require a thorough review and reassessment to incorporate AR/VR specific security and privacy policies and regulations.

Blockchain ensures interoperability and manages virtual transactions and digital asset ownership between sub-metaverses. Truong, Le & Niyato (2023) perform a study on the role of blockchain technology in the metaverse. The research emphasizes the importance of blockchain technology to enhance the security of a decentralized metaverse environment. The analysis highlights the challenges in incorporating blockchain technology into the metaverse infrastructure. One study (Duan et al., 2023) has been conducted regarding the role of cross-chain technology to ensure interoperability among critical blockchain networks including metaverse and the Internet of Things (IoTs). Cross-chain technology involves operational complexities but is crucial to secure virtual assets and transactions. The cross-chain facilitates the exchange of virtual assets and transactions seamlessly between multiple metaverse environments.

### Methodology and contributions

The evolving XR and metaverse ecosystems have introduced a new frontier of immersive threats and are provoking cyber crimes, including sexual abuse, cyberstalking, bullying, avatar rape, *etc*. The insecure XR ecosystem is susceptible to various attacks and vulnerabilities through which potential adversaries can gain unauthorized access to the information of millions of interlinked consumers and applications, like credit card numbers, bank account information, login details, *etc*. The exponential escalation of XR attacks and sophisticated immersive hacking techniques are aggregating strong security concerns among XR stakeholders and cybersecurity experts. The defensive mechanism that have worked so far to protect the digital systems are no longer adequate to secure the complex immersive XR architecture.

No comprehensive security guidelines are available for the metaverse stakeholders and consumers. Extensive XR security features with varying security strengths and development approaches are crucial for embedding privacy, authenticity, integrity, interoperability, and incident reporting in an immersive environment. However, the XR developers, consumers, and investors are unaware of the potential XR threats and security vulnerabilities. Incorporating appropriate security measures while developing XR systems has not been a priority for metaverse and XR developers while competing for the business advantage. It is essential to provide comprehensive security guidelines for the developers to implement various security measures for immersive reality systems.

A panoramic view of defensive mechanisms is presented in this article to ensure authenticity, privacy, integrity, interoperability, virtual forensics, and incident reporting in the metaverse and XR systems. The security significance of defensive approaches to mitigate potential immersive attacks is highlighted. The identified security approaches are then mapped to the development libraries (software development kits (SDKs)/application programming interfaces (APIs)) to determine the corresponding supportive implementable attributes and methods. To the best of our knowledge, this article presents the first defensive model with high-level implementation attributes to counter potential threats of immersive reality systems. Furthermore, the international security standards, government strategies, and global contributions aligned with the metaverse and XR systems are explored. A statistical analysis is conducted to evaluate the quality and effectiveness of available SDKs and APIs to support the implementation of XR security features. Security assessment scores are derived to evaluate the security posture of prominent XR systems. Finally, recommendations are proposed to enhance the security strength of XR systems.

The available resources address a subset of XR security concerns or discuss the XR defensive mechanisms related to a particular XR security weakness. In this article, a thorough research is conducted based on expert insights, technology screening, online resources including web blogs, book chapters, conferences, and published articles from Elsevier, MDPI, NIST, IEEE, ACM, Springer, and Science Direct. This research has identified the XR defensive mechanism from 2017 till date (Nov 2024). The research sources were selected based on their relevance to the proposed XR defensive model. The emerging XR threats were identified and classified, and for each classified threat, potential defensive features were mapped, categorized, and evaluated based on their applicability and effectiveness. The defensive features are selected and categorized to ensure privacy, authenticity, integrity, interoperability, and forensics. Subsequently, XR development resources including SDKs, APIs, plugins, libraries, and toolkits were filtered by prioritizing the defensive capabilities. From these resources, the implementable security attributes were extracted to support the integration of the proposed defensive features in XR systems.

### Paper organization

The remaining article is organized as follows. A defensive model is presented in ‘XR Defensive Model’, to enhance the authenticity, privacy, integrity, interoperability, virtual forensics, and incident reporting in an immersive environment. This section highlights the mitigation capabilities of various defensive mechanisms against potential immersive threats. Furthermore, high-level implementation attributes and functions are determined to support the embedding of corresponding security features. The security analysis and results are discussed in ‘Statistical Analysis and Results’ with an elaboration on defensive features adopted by prominent metaverse and XR industries. The international XR security standards, government strategies, and global contributions are elaborated in ‘XR Security Standards, Regulations and Government Strate Gies’, emphasizing the security and privacy of virtual users. In ‘XR security recommendations’, significant security features for sensitive XR domains and a checklist of security measures are advised to safeguard the XR system. Limitations and future research directions are discussed in ‘Limitations and Future Direction’. Finally, the ‘Conclusion’ summarizes the key findings of our research.

## XR defensive model

**Figure 1:**
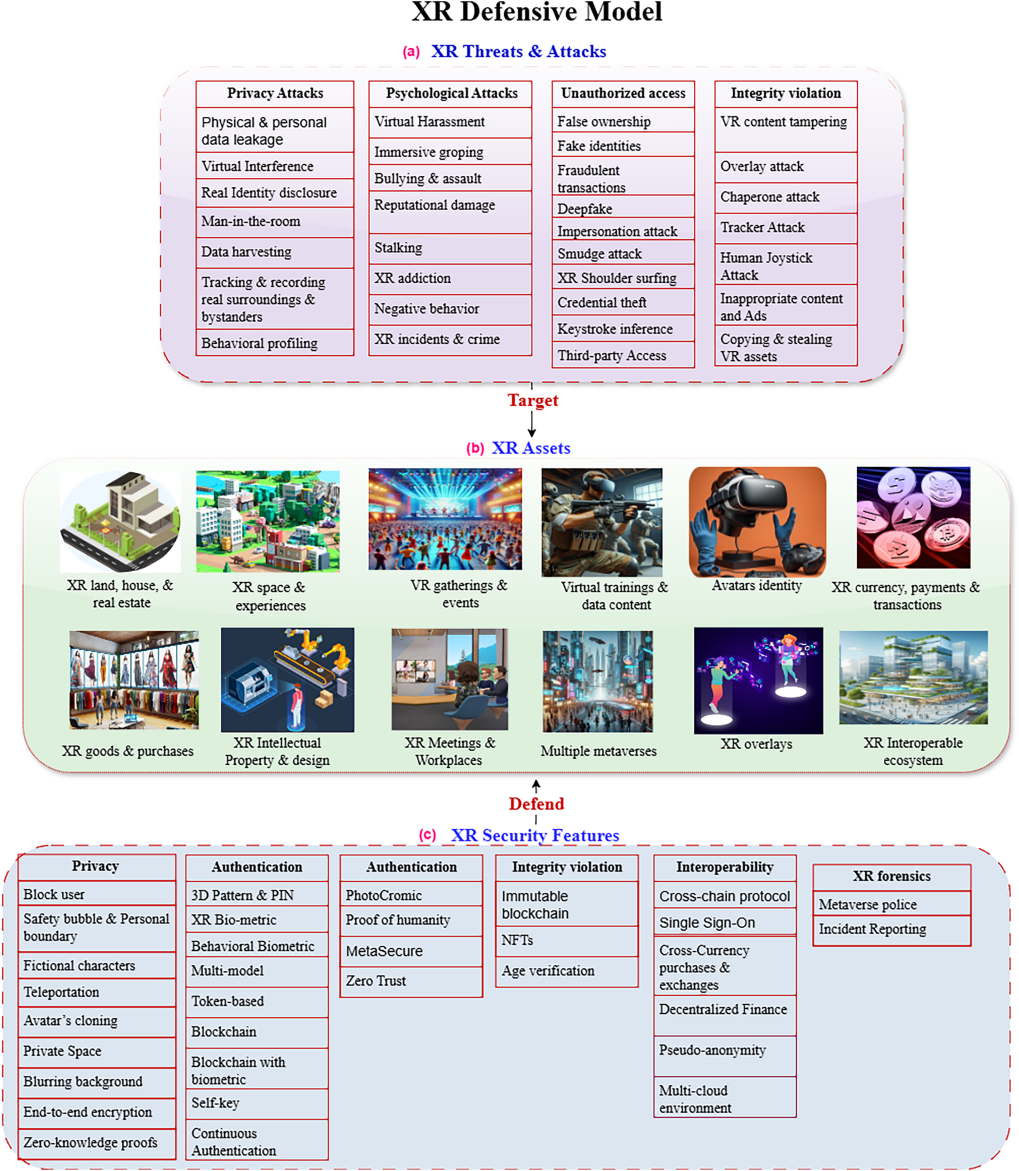
XR defensive model.

With the rapid development of metaverse and immersive reality systems, various potential threats, novel XR attacks, and immersive cyber crimes have emerged that exploit zero-day vulnerabilities. Our article provides a defensive model by highlighting the strengths and mitigation capabilities of controls against potential XR attacks to protect the virtual assets and metaverse ecosystem. This section presents the defensive features to enhance the authenticity, privacy, integrity, interoperability, virtual forensics, and incident reporting in the metaverse and XR systems. The identified defensive features are then mapped to the XR development libraries and toolkits to extract the supportive implementation attributes and functions. Figure 1 shows high-level architecture of XR defensive model presented in this article. Figure 1A highlights the potential security threats targeting immersive experiences, Fig. 1B shows the key virtual assets targeted by XR attacks, and Fig. 1C outlines the defensive features to protect XR ecosystems.

In this section, we illustrate the set of supportive attributes aligned with the development resources to incorporate security features at the source code level. The enumerated XR security features and the corresponding implementable attributes are compiled from various development resources by examining prominent software development kits (SDKs) and application programming interfaces (APIs), libraries, tool kits, plugins, and platforms, including Unity3D (Unity Technologies, 2025), Unreal Engine (Epic Games Inc, 2024), WebXR (Mozilla Foundation, 2023) and more. The set of identified development resources used to implement security features in XR systems are listed in Table 1.

**Table 1:** Identified development resources for implementing defensive features in XR (“XR Defensive Model”).

SDKs	APIs	Plugins	Libraries	Toolkits
• OpenXR	• OVRBoundary	• SteamVR	• Web3.js	• Unity XR interaction
• WebXR	• BiometricPrompt	• ARCore	• Ethers.js	• Unity XR Boundary
• Unity3D	• uPort	• OpenCV	• ZoKrates	• Microsoft Mixed Reality
• Unreal Engine	• Veridium		• Sensor Fusion	• Virtual Reality toolkit
• Oculus platform	• Ethereum			• Vircadia
• Steamworks	• High Fidelity			• zkSync
• VRChat	• Somnium Space			• STARKs
• Viveport	• Auth0			• Bulletproofs
• Leap Motion	• TypingDNA			• Google Identity
• Oculus Quest Hand Tracking	• UnifyID			• OpenID Connect
• Vive Hand Tracking	• BehavioSec			• EOSIO
• Decentraland	• BioCatch			• Hyperledger Fabric
• SelfKey	• FaceTec			
• Unity multiplayer	• OnfidoFaceTec			
• Uniswap	• iProov			
• SushiSwap				
• Neos VR				
• Agora RTC				
• Matrix				
• NeuroSky MindWave				
• Blockstack				

### Privacy

**Figure 2:**
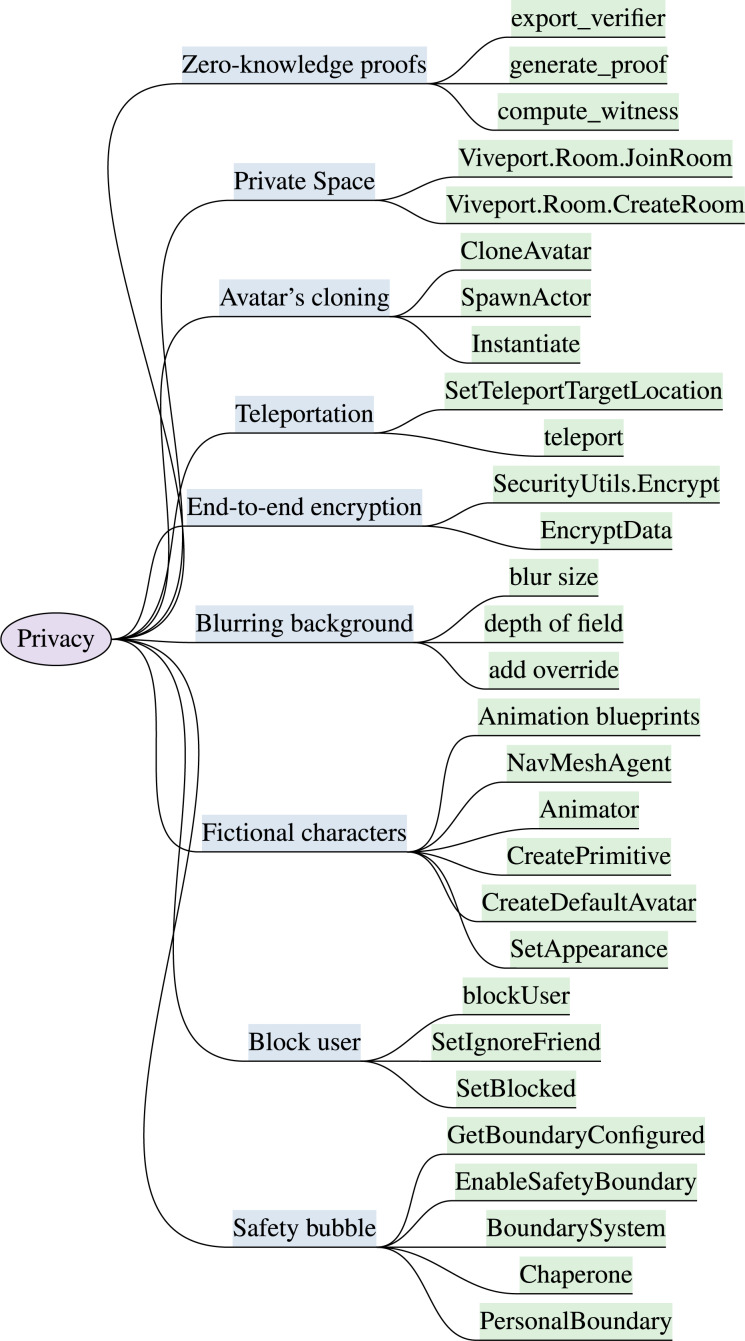
Identified privacy features & implementation attributes in XR (“Privacy”).

Metaverse operates on users’ data to mimic reality in an immersive environment. The conventional privacy approaches are unable to provide privacy in immersive reality systems. The front-facing cameras and motion sensors available in most extended reality (XR) head-mounted displays (HMDs) can be exploited by attackers to stream the video feed, grant access to the user’s physical surroundings, and record speech-associated facial gestures. The illegal disclosure of user’s physical and personal information leads to data breaches and users’ behavioral profiling in immersive reality. These privacy challenges emphasize the requirement for privacy-ensuring techniques to safeguard against immersive crimes effectively. This section presents privacy features with security significance and mitigation capabilities against potential threats. The identified privacy enhancing features for immersive reality with the implementation attributes are discussed, and the key attributes are outlined in Fig. 2. The section highlights are available in Table 2.

**Table 2:** Privacy features and mitigation in XR.

No.	Privacy features	Significance	Prevents
1	Blockuser	Block unwanted virtual participants	Negative interaction
2	Safety bubble & Personal boundary	Maintain safe distance in virtual interaction	• Immersive groping
			• Bullying
			• Virtual harassment
			• Immersive assault
3	Fictional character	Hide real-world identity & Maintaining anonymity	• Doxxing
			• Virtual discrimination
			• Profiling
4	Teleportation	Off-track observers in immersive world	• Stalking
			• Tracking
			• Behavioral profiling
			• Harassers
5	Avatar’s cloning	Disguise user’s true identity	• Stalking
			• Tracking
			• Profiling
6	Private Space	Private copy of potion of virtual space	• Unauthorized access to virtual space
			• Unwanted immersive interactions
			• Impersonation
			• Behavioral profiling
			• Harassers
7	Blurring background	Bystander privacy & anonymity	• Doxxing
			• Bystander exposure
			• Environmental profiling
			• Background surveillance
8	End-to-end encryption	Ensure privacy of virtual & immersive content	• Virtual interference
			• Man-in-the-room attack
			• Unauthorized access to virtual transactions
9	Zero-knowledge proofs (ZKPs)	• Identity verification	• Identity, Information &
		• Transaction privacy & anonymity	Transaction disclosure
		• Authenticity	• Unauthorized access
		• Data verification	• Identity & Credential theft
		• Trust in virtual interaction	• Tampering
			• Background surveillance

***Block user:*** Metaverse users can block unwanted virtual participants from their virtual space by clicking on their avatar and selecting the block option (Tom Blackstone, 2022). Blocking another user is easy to embed because it is a common feature in most of the XR and metaverse API. To enable the feature of blocking users in an XR system, the function of blockUser is available in Decentraland User API (Decentraland, 2023a), High Fidelity User API (High Fidelity Inc, 2023a), VRChat User API (VRChat Inc, 2025) and Somnium Space SDK (Somnium Space, 2025). This feature is also available in Oculus platform SDK (Meta Quest, 2023a) and Steamworks (Steam, 2023) using functions of SetBlocked and SetIgnoreFriend. In the metaverse and XR system, the fundamental level of privacy is achieved by allowing users to block unwanted person’s avatar in his virtual space to mitigate negative interactions.

***Safety bubble & Personal boundary:*** XR users can create a boundary or a safe distance while virtually interacting with other users to prevent immersive groping and virtual harassment (Tom Blackstone, 2022). Meta Facebook has introduced a feature to maintain a 4-foot boundary between users. The feature of Safety bubble & Personal boundary is a crucial protection mechanism to ensure privacy in public VR spaces. If a user feels terrified from immersive surroundings, he can enable the safety bubble or personal boundary option to prevent getting hurt by anyone. This feature aids users in preventing immersive assault, groping and bullying. The subsequent methods like PersonalBoundary, Chaperone, BoundarySystem, EnableSafetyBoundary, GetBoundaryConfigured of Oculus SDK (Meta Quest, 2023a), SteamVR (Valve Corporation, 2023), Microsoft Mixed Reality Toolkit (MRTK) (Microsoft, 2024a), Unity XR interaction toolkit (Unity XR Boundary System) (Unity Technologies, 2025), and OVRBoundary API (Meta, 2022b) can be integrated to maintain a safe distance between participants.

***Fictional characters:*** Certain XR platforms allow users to hide their real-world identity and physical appearance while interacting in the metaverse. The users can create avatars with entirely dissimilar features from their actual characteristics. Maintaining anonymity in an immersive environment is essential and can be achieved using features of fictional characters to avoid misuse of personal information, identity theft, doxxing and discrimination. XR APIs support creation and customization of avatar which hide users physical appearance in virtual world. The supported functions like CreateDefaultAvatar, CreatePrimitive are available in Neos VR SDK (Neos, 2024) and Unity XR Interaction Toolkit (Unity Technologies, 2025) respectively. The attributes involved in the implementation of avatar’s fictional characters also involve Animator component for personality traits, NavMeshAgent to generate predefined behaviors in Unity3D, and animation blueprints option to generate and control animation states.

***Teleportation:*** Teleportation facilitates XR clients to transport their avatars to another immersive land (Abbate et al., 2023). This feature helps clients off-track observers in immersive world, increase difficulty for attacker to create user’s profile based on his virtual interaction, and behavior analysis. The feature prevent stalking, tracking, behavioral profiling, and avoid harassers. The APIs including Unity XR interaction toolkit (Unity Technologies, 2025), SteamVR plugin (Valve Corporation, 2023), Mixed Reality toolkit (MRTK) (Microsoft, 2024a), VRTK (Virtual Reality toolkit) (VRTK/Sysdia Solutions Ltd, 2024) provide component and plugins for the implementation of teleportation functionality like Teleportation provider, Locomotion system, SteamVR teleport, Teleport system, VRTK_Teleport respectively. SetTeleportTargetLocation function in VR expansion plugin (Aster94, 2023b) of Unreal Engine (Epic Games Inc, 2024), teleport method in SteamVR and teleportation module in Vive Input Utility (VIU) (Vive Developers, 2023) of Unity, can also be adopted for the development of teleportation module in XR system.

***Avatar’s cloning:*** XR users can create their avatar’s clones that are identical in virtual appearances to confuse XR stalkers, misdirect attackers, and aid in losing track of the user’s actual avatar (Zelenyanszki et al., 2023). The feature disguises the user’s true identity. To implement this feature Unity (Unity Technologies, 2025) and StreamVR (Valve Corporation, 2023) support multiple avatar instances using Instantiate function, Unreal Engine (Epic Games Inc, 2024) support actor duplication using SpawnActor method, High Fidelity API (High Fidelity Inc, 2023a) creates multiple instances of avatars by calling method of addEntity, JanusXR (Baicoianu & Singh, 2024) support cloning entities and Microsoft Mixed Reality Toolkit (MRTK) (Microsoft, 2024a) provide object duplication. The CloneAvatar method of SteamVR plugin, VRChat (VRChat Inc, 2025), and Ready Player Me SDK (Ready Player Me Inc, 2023), can be accommodated to achieve the feature of virtual avatar cloning.

***Private Space:*** XR users can create or demand a private copy of a portion of the virtual world, where only the user and their invited friends can live (Park, Ahn & Lee, 2023). This feature protects users from unauthorized access to their virtual space, unwanted immersive interactions, harassers, data harvesting, impersonation attacks, and behavioral profiling. UnityXR enables custom scenes, layers, and permission settings, while Unreal Engine (Epic Games Inc, 2024) provides level streaming and player-specific permissions to generate private isolated virtual experiences. High Fidelity (High Fidelity Inc, 2023a) offers spatial audio zones and Microsoft Mixed Reality Toolkit (MRTK) (Microsoft, 2024a) supports spatial anchors and bounding boxes. Additionally, the configuration settings of private spaces like JoinOrCreateRoom in Photon Unity Networking (PUN) (Unity Technologies, 2023), Viveport.Room.CreateRoom and Viveport.Room.JoinRoom in Viveport SDK (Vive Developers, 2023) can be incorporated to create a private immersive experience.

***Blurring background:*** The feature of blurring background is important to protect surroundings and bystander privacy. The user intentionally blur the background of recordings, to protect bystanders’ information, environmental profiling, and to degrade the quality of capture related to users’ physical background and surroundings (Dimiccoli, Marín & Thomaz, 2018). No built in single method is available for this feature, the post-processing actions of the camera must be configured to blur background effects using attributes available in Unity and Unreal Engine (Epic Games Inc, 2024) such as post-processing stack, add override, depth of field and blur size. The developers can also integrate ARCameraBackground and MonoBehaviour components available in Unity3d Engine (Unity Technologies, 2025).

***End-to-end encryption (E2EE):*** User data must be end-to-end encrypted to safeguard against interference by other virtual participants in metaverse, man-in-the-room attack (MITR), and even the systems and XR gadgets should be prohibited to access the users data and should be fully private. The implementation of E2EE feature in immersive space is challenging because XR devices are resource constrained, require high interactivity, spontaneous responses with low latency (Chen et al., 2024). The XR headsets and gadgets have limited processing power and the execution of E2EE is computationally intensive tasks, introduces further delay and consume additional battery power. Key generation and distribution to perform encryption in decentralized metaverse is also challenging (Bentotahewa et al., 2023). Very limited XR systems support this feature, Meta’s Horizon Worlds (Meta, 2024a), and Decentraland (Decentraland, 2023b) claims to support limited encryption to secure users data and virtual communications only. This feature defend against numerous attacks including virtual interception, man-in-the-room attack (MITR), and unauthorized access to virtual transactions. Agora RTC SDK (Agora Inc, 2024) provide real-time encryption for audio and video communication using methods like EnableEncryption and SetEncryptionMode. Matrix SDK (The Matrix.org Foundation, 2024) support E2EE for decentralized communication protocol using functions like encryptMessage. Vircadia (DigiSomni, 2024) open-source metaverse platform which support E2EE using functions like addPacketTypeHandler. Oculus SDK (Meta Quest, 2023a) support secure messaging and data transmission between Oculus users. The Crypto library functions like EncryptData in Unreal Engine (Epic Games Inc, 2024) and SecurityUtils.Encrypt by unity 3D (Unity Technologies, 2025) also support encryption.

***Zero-knowledge proofs (ZKPs)***: The cryptographic technique used to conduct identity verification while maintaining transaction privacy, authenticity, data verification and validity of any information without revealing any knowledge is known as zero-knowledge proofs. Zero-knowledge proofs in immersive setting is utilized to establish trust among virtual interactions while preserving privacy, and establishes transactions with shielded addresses. The virtual transactions like sender, amount, and receiver details remain encrypted but verifiable using this feature. The ZKPs allow virtual users to establish trust in a decentralized manner. ZKPs defend against identity, information and transaction disclosure. Furthermore, the technique mitigate unauthorized access, credential theft, tampering and identity theft (Chen et al., 2024). Few metaverse and XR applications support this feature including Decentraland (Decentraland, 2023a), Somnium Space (Somnium Space, 2025) and High Fidelity (High Fidelity Inc, 2023a) for transaction verification. ZoKrates (ZoK, 2020) toolbox for zk-SNARKs (zero-knowledge succinct non-interactive arguments of knowledge) on Ethereum provide support for implementing privacy-preserving interactions, by creating and verifying zero-knowledge proofs on Ethereum and other blockchain platforms. Zcash (Wilcox-O’Hearn & Green, 2023) is another privacy-focused cryptocurrency that employs zero-knowledge proofs to enable anonymous transactions. The subsequent methods compute-witness, generate-proof, export-verifier can be adapted to compute the witness for the given inputs, generate a zero-knowledge proof, and export the verification key. The development resources such as zkSync (zkSync, 2024b, 2024a), STARKs (Scalable Transparent ARguments of Knowledge) (StarkWare, 2024), and Bulletproofs (Security, 2024) also support implementation of ZKPs.

### Authentication

Identity fraud in the metaverse can cause substantial financial and reputation damage to stakeholders. Fake identities can be generated in XR and there is currently no trust mechanism exists to verify that a person’s avatar is interacting with the same virtual party which he is assuming. Ensuring trust between virtual users and collaborating parties in the immersive reality is challenging. The users in the metaverse communicate with the digital avatars, and the compromised credentials may lead to deepfake attacks. DeepFake (Wu, Hui & Zhou, 2023) technology in XR allows users to manipulate and replace their digital appearance with another person.

**Figure 3:**
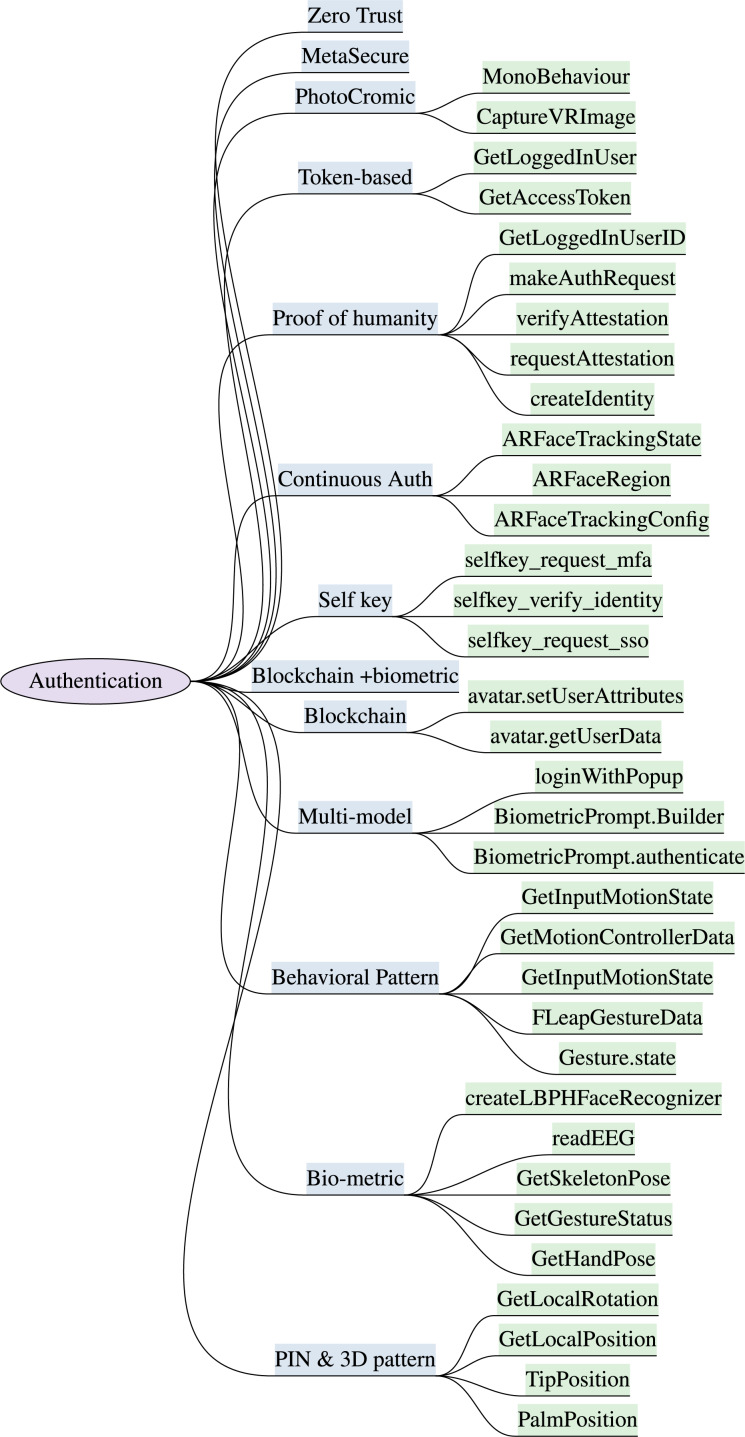
Identified authentication features & implementation attributes in XR systems (“Authentication”).

Traditional authentication methods of login through PIN and password *via* keyboard are prone to numerous attacks and vulnerabilities in XR. The head-mounted displays (HMDs) of XR blocks user’s physical view are susceptible to attacks such as shoulder surfing and keystroke inference attacks. The virtual keyboard is also vulnerable to diverse attacks (Kürtünlüoğlu, Akdik & Karaarslan, 2022) including wireless signal-based, video-based, and malware-based attacks. Authentication techniques for the XR and metaverse consumers must be scalable, decentralized, resilient to node damage, and interoperable across multiple sub metaverse. The XR authentication model must consume low storage/memory and computing capability. The identified XR authentication schemes with the supportive implementable attributes are discussed below and are depicted in Fig. 3. The security requirements and resiliency of authentication features to counter potential threats are elaborated in this section and outlined in Tables 3 and 4. Table 3 highlights the significance of various biometric, behavioral, and continuous authentication features for immersive XR sessions. The Table 4 presents blockchain-based authentication features, with their security significance and mitigation capabilities to counter XR security threats.

**Table 3:** XR biometric, behavioral & continuous authentication features.

No.	Authentication features	Significance	Prevents
1	3D pattern & Password	Harder to mimic	• Brute-force
			• Smudge attacks
			• Password cracking
2	Bio-metric	• Quicker	• Shoulder surfing
		• Unforgettable	• Tampering
		• Harder to steal & reuse than 3D pin	
3	Behavioral biometric	• Uniqueness	• Brute-force
		• Harder to steal, forge & reuse than 3D password and biometric	• Shoulder surfing
			• Tampering
			• Impersonation
			• Smudge attacks
			• Account takeover frauds
			• Social engineering
			• Man-in-the-middle
			• Credential theft
			• Replay attacks
4	Multi-model authentication	Enhanced strength, attackers need to bypass multiple controls	• Brute-force
			• Smudge attacks
			• Password cracking
			• Shoulder surfing
			• Tampering
			• Impersonation
			• Account takeover frauds
			• Credential theft
			• Social engineering
5	Continuous authentication	Ensure human identity	• Deep fakes
			• Impersonation
			• Identity theft
			• Avatar spoofing
			• Session hijacking
			• Replay attacks
6	PhotoCromic	• Creating, managing & verifying identities	• Unauthorized access
		• Photochromic materials provide an	• Identity theft
		additional layer of security	• Identity Spoofing
		• Biometric proof of life	• Tampering
		• Government-backed identity verification	• Credential exposure
		• Social media attestations	• Deepfake
			• Sybil attack
			• Unauthorized access to virtual identities and assets
			• Replay attacks
7	Proof of humanity	• Data privacy	• Deepfake
		• Digital footprint	• Bullying
		• Anonymity	• Immersive frauds
		• Interoperability	• Identity theft
		• Trust	• Impersonation
			• Proof of identity
			• Ownership & transaction verification
8	MetaSecure	• User-defined keys	• Unauthorized access to virtual assets
		• Seamless key management	• Identity theft
		• Seamless access control	• Bullying
		• Triple layer authentication (device	• Cyberstalking
		attestation, security key & facial recognition)	• Transaction Forgery
		• Secure virtual assets, Immersive identities,	• Replay
		and financial transactions	• Impersonation
9	Zero trust	• Identity verification & Continuous authentication	• Unauthorized access
		• Integrity & Cross-platform interaction	• Identity theft
		• Identity verification & Authorization	• Tampering
		• Trust	• Data exfiltration

**Table 4:** XR block-chain oriented authentication features.

No.	Authentication features	Significance	Prevents
10	Token-based	• Seamless & inter-operable	• Credential reuse & theft
		• Reduce attack surface	• Session hijacking
			• Token Forgery
			• Replay
			• Brute force
11	Blockchain	• Proof of digital identities	• Impersonation
		• Virtual assets ownership	• False acquisition & Ownership
		• Interoperability	• Data tampering
			• Replay
			• Unauthorized access to virtual assets
12	Blockchain+biometric	• Proof of digital identities	• Impersonation
		• Signed transaction	• Identity theft
		• Virtual assets ownership	• Identity theft
		• Interoperability	• False acquisition & Unauthorized access to virtual assets & transactions
			• Data tampering
			• Transaction forgery
			• Replay
13	SelfKey	• Self-sovereign identity management system	• Credential reuse
		• Proof-of-identification	• Identity theft
		• Seamless login	• Unauthorized access to virtual assets and transactions
		• Data privacy (store information locally)	• Data breaches
		• User ownership (control over personal data)	• Credential exposure
			• Sybil attacks

***3D Pattern & Password:*** The most commonly practiced authentication mechanism in the virtual world is by logging through a 3D pin, pattern, and password (Kürtünlüoğlu, Akdik & Karaarslan, 2022). The user performs a sequence of interactions in the virtual environment using VR controllers, and the 3D hand movements are tracked to authenticate the XR users. This feature is more resistant against smudge attacks and brute force and is more difficult to crack than the knowledge-based password schemes because there is no predefined procedure for 3D patterns (Gadhwal, 2024). Incorporating personalized 3D space for authentication makes it much harder to mimic passwords in virtual environments. This method is susceptible to shoulder surfing, as metaverse gadgets completely block out users’ physical surroundings, allowing attackers to observe users’ 3D movements and potentially steal virtual credentials. Predicting login details becomes straightforward by monitoring users’ hand movements. This feature is comparatively easier to implement than the remaining approaches discussed below. It requires no additional cost and accessories, and the probability of attack success is very high, with little effort. This authentication technique lacks interoperability, requiring users to memorize passwords for each metaverse platform. The SDKs deal with the tracking movements such as leap motion controller (Ultraleap, 2023) and unity XR input (Unity Technologies, 2025), with the aligned methods comprised of Hand.PalmPosition, Finger.TipPosition in Leap Motion SDK and InputTracking.GetLocalPosition, InputTracking.GetLocalRotation of Unity XR Input. Oculus SDK also provides input functions such as OVRInput.Get and OVRHand.GetFingerIsPinching for the 3D pattern.

***Bio-metric Authentication:*** The metaverse introduces devices and sensors that provide a range of biometric verification and sensing features for identity management, including Electroencephalography (EEG) (Anastasaki et al., 2023), iris (Wang, Li & Yan, 2023), *etc*. Biometric authentication in the metaverse is more reliable than 3D passwords and is notably quicker, unforgettable, and harder to steal and reuse (Kürtünlüoğlu, Akdik & Karaarslan, 2022). It requires additional biometric hardware like EEG headsets and iris scanners, incurring extra costs for buyers and providers to store and verify biometric credentials (Cointelegraph, 2023). This technique resists tampering and shoulder-surfing attacks as the authentication information is not directly exposed to attackers, compared to a 3D pin in a virtual environment. NeuroSky MindWave SDK provides readEEG and authenticates functions to capture EEG signals and OpenCV supports createLBPHFaceRecognizer and recognize functions for facial recognition and behavioral analysis. Furthermore, the methods for implementing biometric authentication in XR include Oculus.HandTracking.Hands.GetHandPose and Oculus.HandTracking.Hands.GetSkeletonPose are available in Oculus Quest Hand Tracking SDK (Meta, 2022b), and ViveHandTracking.GetHandPose and ViveHandTracking.GetGestureStatus are provided in Vive Hand Tracking SDK (Vive Developers, 2023).

***Behavioral pattern & Behavioral biometric***: This XR authentication scheme is based on the user’s gesture or behavior analysis which tracks user movements, spatial interactions, navigation style, object manipulation, and gesture recognition with the help of integrated sensors in a virtual environment (Cointelegraph, 2023). Cracking behavioral patterns requires more diligence, monitoring, and time and is harder to steal, forge, and reuse than the 3D password and biometric authentication (Chow et al., 2022; Kim et al., 2023). Behavioral analysis predicts the slightly strange behavior of unauthorized users and their login attempts can be blocked in immersive reality. The behavioral biometrics feature also defends against brute force, shoulder surfing, tampering, impersonation, and smudge attacks (Cheng, Chen & Han, 2023). The feature of behavioral biometric will prevent account takeover frauds, social engineering, man-in-the-middle, credential theft, and replay attacks (Incognia, 2024; PLURILOCK, 2024). The attributes for behavior analysis can be incorporated into the XR systems by importing the following SDKs, such as Unreal Engine (Epic Games Inc, 2024), unity 3D (Unity Technologies, 2025), Leap Motion SDK (Ultraleap, 2023), and Sensor Fusion Libraries (Aster94, 2023a). The key attributes for behavioral analysis include MonoBehaviour and Leap Motion’s Gesture classes of Leap Motion SDK, Gesture.Type and Gesture.state properties of unity3D. Similarly, FLeapGestureData::state, FLeapGestureData::Type, GetMotionControllerData, and GetInputMotionState, of Unreal Engine can be used to access the gesture information and profile matching. Azure Cognitive Services and BehavioSec (Microsoft, 2024b; LexisNexis Risk Solutions, 2024) also provide methods for behavioral analysis and anomaly detection.

***Multi-model Authentication:*** This technique authenticates metaverse users by verifying a combination of parameters including PIN/password and bio-metric. Users are required to enter a 3D pattern or password with some biometric behaviors for multi-model Authentication. Employing a multi-model authentication scheme enhances security strength and reliability, and defends against the security challenges faced by single authentication techniques, as attackers must exert additional efforts to overcome multiple controls (Kürtünlüoğlu, Akdik & Karaarslan, 2022). The feature can be established by integrating multiple SDKs/APIs to support multiple authentication attributes. The BiometricPrompt API (Android Developers, 2025) supports features to enable authentication using biometric modalities like fingerprint, face, or iris. The major functions comprise of BiometricPrompt.Builder and BiometricPrompt.authenticate. The Auth0 (Auth0, 2024) SDK, Microsoft Azure Mixed Reality Services (Microsoft, 2024c), and Google Identity Platform (Google, 2024a) also support various functions to implement the multi-model approach in XR.

***Continuous Authentication:*** In XR and the metaverse environment, continuous authentication identifies the unique biometric features of the human body, especially the human face, captured from images, video, or through the physical activities of consumers with high precision. Continuous identity verification of XR users while wearing their headset ensures that the person using the headset is still the identified and authenticated person (Takahashi, 2022). Continuous biometric verification and continuous behavioral bio-metric (Cointelegraph, 2023) are recommended for metaverse users to ensure the availability of human identity and to prevent potential threats such as deep fakes. This feature facilitates XR users with a seamless, non-intrusive experience and enhances scalability. The XR users don’t need to remember multiple passwords using this feature. Continuous biometric authentication further increases the difficulty level for attackers to mimic legitimate users’ behavior over an extended period. The likelihood of an attack success to bypass continuous authentication technique is 1 in 1,000,000 attempts (HKT Enterprise, 2022). The slight anonymous behavior of users will terminate the session. Furthermore, the feature defends against identity spoofing, credential theft, impersonation, and replay attacks on ongoing identity verification in the XR environment (Cheng, Chen & Han, 2023; Yang et al., 2023; Dahad, 2023). Multiple APIs including TypingDNA, UnifyID, BehavioSec, and BioCatch (TypingDNA, 2024; BioCatch, 2024; LexisNexis Risk Solutions, 2024) support continuous authentication by analyzing users’ behavior and biometrics. The Unity 3D (Unity Technologies, 2025) SDK, ARCore (Google Developers, 2023), and OpenCV (doxygen, 2023) plugins deal with the capabilities of image processing and face recognition in the metaverse. The key functions include ARFaceTrackingConfig, ARFaceRegion, and ARFaceTrackingState.

***MetaSecure:*** MetaSecure (Sethuraman et al., 2023) is a password-less authentication technique for the metaverse to secure virtual assets, immersive identities, and financial transactions. It is a blend of three distinct methodologies, including device attestation, facial recognition, and physical security keys or smartcards. The physical security keys are used with the device attestation and facial recognition to secure the metaverse. MetaSecure protects against potential threats of unauthorized access to virtual assets, identity theft, bullying, cyberstalking, transaction forgery, impersonation, and replay. APIs and SDKs are available to support these authentication features including SafetyNet Attestation API and Play Integrity API (Google, 2024b) to provide device attestation, Microsoft Azure Face API (Microsoft, 2024b) to support facial recognition, and WebAuthn API facilitate physical security keys. These features can be integrated to embed the MetaSecure authentication scheme in XR systems. The Veridium API (Apideck, 2023) also claims to provide password-less authentication in the virtual world

***PhotoChromic:*** PhotoCromic is a shared protocol for creating and managing identities on blockchain based XR networks, software services, and decentralized apps (PhotoChromic, 2022). It aggregates a biometric proof of life, government-backed identity verification, social media attestations, and unique personal attributes into a single blockchain asset, used for identity verification in XR. It serves as a biomimetically managed model of Self-Sovereign Identity (SSI) on the Blockchain. The PhotoCromic scheme serves as an image-based authentication in the immersive environment that utilizes photochromic materials to verify the user’s authenticity. The photochromic substances modify color and opacity in response to particular stimuli *e.g*., light or UV radiation. The unique properties of photochromic materials provide an additional layer of security and resist session hijacking, replay, and impersonation attacks. PhotoCromic ID is a privacy-preserving, portable identity and a digital passport used to travel and transact between multiple metaverse. This feature prevents XR users from identity theft, spoofing, tampering, credential loss, deepfake, sybil attacks, unauthorized access to virtual identities and assets, and replay attacks. The APIs including FaceTec, OnfidoFaceTec, iProov (FaceTec, 2024; Onfido, 2024b; iProov, 2024) are offering support for photochromic features that could be integrated to scan biometrics, authenticate faces, and perform liveness checks. For PhotoCromic implementation, the developers can also import image processing functions like CaptureVRImage and MonoBehaviour, which are available in *OpenCvSharp.Unity* (doxygen, 2023).

***Proof of humanity:*** The Proof of Humanity is a blockchain-based authentication mechanism that identifies users without comparing any of their unique information, like a national ID card or location, and maintains the digital footprint of users. Moreover, the technique facilitates interoperability, identity verification, and trust while interacting in the immersive reality. The identity of users is proven with a video and a crypto wallet URL without any knowledge or biometric matching (VentureBeat, 2022). It maintains data privacy, and anonymity and prevents credential leakage. It’s a form of self-sovereign identity (SSI) (decentralized identity) which gives users full ownership of their digital identity instead of third-party. Proof of Humanity safeguards users from credential theft based on *SSI*. The feature protects XR users against cyber attacks including impersonation, deepfake, bullying, immersive frauds, and identity theft. The Unity XR Interaction Manager, Unreal Engine and Microsoft Mixed Reality Toolkit (MRTK) supports the development of Proof of Humanity. Security functions used to implement this technique include createIdentity, requestAttestation, verifyAttestation using uPort API (uPort, 2024), makeAuthRequest from Blockstack (Blockstack, 2024) SDK, and OculusIdentity.GetLoggedInUserID provided in Oculus SDK (Meta Quest, 2023a).

***Zero trust:*** Zero trust model operates on the concept of ”never trust, always verify” in the metaverse and XR domain. It emphasizes identity verification and continuous authentication in a virtual environment to preserve integrity and cross-platform interactions (Sun et al., 2022). Zero-trust architecture in immersive systems incorporates a combination of security techniques to ensure secure and trusted interactions within the virtual world. Zero-trust assumes no inherent trust and requires verification and authorization of all users, systems, and integrated devices. Zero-trust ensures that unauthorized users are kept out of the system and is the most robust technique to reduce the XR security risks. The zero-trust architecture is crucial to counter critical novel immersive attacks, including unauthorized access, identity theft, tampering, and data exfiltration. Okta, Auth0, Azure AD, and Google Cloud Identity (Okta; Auth0, 2024; Microsoft, 2024d; Google, 2024a) facilitates developers to implement zero-trust model in XR systems. The key attributes of Okta include signInWithCredentials, acquireTokenInteractive (Microsoft, 2024e) available in Azure AD, and signInWithIdToken (Okta, 2024) in Auth0.

***Token-based authentication:*** In a token-based authentication scheme (Durr, 2022), the users can seamlessly browse and use different platforms without reentering login details. The users enter their credentials once, and the system initializes a digitally encrypted token that authenticates the clients and allows access to the resource for a particular session. The token could be hardware, connected to the system like a USB or a smart card, or a software-based token like a JSON (JavaScript Object Notation) web token. The Token-based authentication feature facilitates XR users by offering a seamless and inter-operable metaverse experience. The token is destroyed with the session expiry, short-lived tokens reduce the attack surface and minimize the risk of unauthorized access and credential theft. This feature resists session hijacking, replay, and brute forceattempts. The tokens are used with standardized protocols like OAuth2 and OpenID Connect and they support scalability and integrity with various XR devices in a metaverse environment. Oculus.Platform.Users.GetAccessToken and GetLoggedInUser functions of Oculus SDK (Meta Quest, 2023a) can also be integrated to obtain the user’s login information and access token request.

***Blockchain:*** The user’s digital identity and proof of ownership of their virtual transactions are essential for a stable metaverse economy. The blockchain-based identity verification mechanism is considered the most secure solution for an integrated metaverse environment (Sun et al., 2022). Furthermore, blockchain provides interoperability among multiple platforms based on decentralized infrastructure and incorporates asymmetric encryption and hash functions to ensure data security for XR infrastructure. Blockchain facilitates XR stakeholders by granting proof of digital identities and virtual assets ownership, using crypto wallet with their respective private keys. The blockchain and decentralized authentication features protect immersive identities against various XR attacks. Blockchain implementation is resource-intensive and expensive, introduces latency, and impacts the scalability of the metaverse environment (Nagar, 2022). Blockchain features prevent false ownership, data tampering, replay, server spoofing, and unauthorized access to virtual assets (Kim et al., 2023). Decentraland (Decentraland, 2023a), Ethereum (Ethereum, 2023), Web3.js (Meta, 2022a), Azure Blockchain Service (Russinovich, 2024), and Cryptovoxels (Crunchbase Inc, 2023) SDKs support the integration of blockchain features in the XR systems. The available supportive development attributes of Decentraland SDK include avatar.getUserData(address) and avatar.setUserAttributes(attributes).

***Blockchain with biometric:*** Unique biometric features are integrated with blockchain to secure virtual assets and digital identities in the metaverse. Biometric properties serve as a basis for the generation of cryptographic public-private key pairs for user authentication. These keys will serve as proof of identity and to digitally sign transactions in the metaverse. This feature prevents identity theft, false ownership, data tampering, replay, server spoofing, impersonation, and unauthorized access (Kim et al., 2023). The implementation attributes for blockchain and biometric features are already illustrated earlier in this section. The digital identity based on key pairs is considered a more robust security mechanism to preserve users’ authenticity in the metaverse and XR (Sun et al., 2022).

***Selfkey:*** SelfKey is a decentralized, blockchain-based self-sovereign identity management system that permits XR users to control their digital identity, virtual assets, and transactions. SelfKey is a noncustodial wallet that protects XR users’ identity and data privacy by storing the personal information locally on the consumer’s system (Joseph, 2021). The SelfKey (KEY) blockchain-based self-sovereign identity system is built on the Ethereum (Ethereum, 2023) and underlying key token mechanism is cryptographically secure (ERC-20 compliant). SelfKey uses a proof-of-identification (POI) (Krishnamohan, 2022) consensus algorithm to grant the metaverse users the right to control their data and protect its access from third-party. It is highly challenging for attackers to steal users’ credentials and identity, built on SelfKey (KEY) (Peterson, 2022). SelfKey SDK (SelfKey Identity Wallet and SelfKey ID APIs) (SelfKey Foundation, 2025) offers a decentralized identity ecosystem, that incorporates identity management and verification features for the metaverse and XR environment. The security features offered by this scheme include identity creation, verification, attestation, and a seamless login experience without separate usernames and passwords. SelfKey facilitates XR users by offering them complete control over their personal data usage, access, and sharing rights. The feature reduces the risk of data breaches, identity theft, credential exposure, and unauthorized access to virtual assets and transactions. Furthermore, the SelfKey platform claims to protect the decentralized metaverse environment from Sybil attacks (SelfKey, 2023b, 2023a). Single sign-on attributes using SelfKey SDK are selfkey_request_sso (for single sign-on), selfkey_verify_identity, selfkey_request_mfa, and selfkey_verify_mfa_code (for multi-factor authentication).

### Integrity

**Figure 4:**
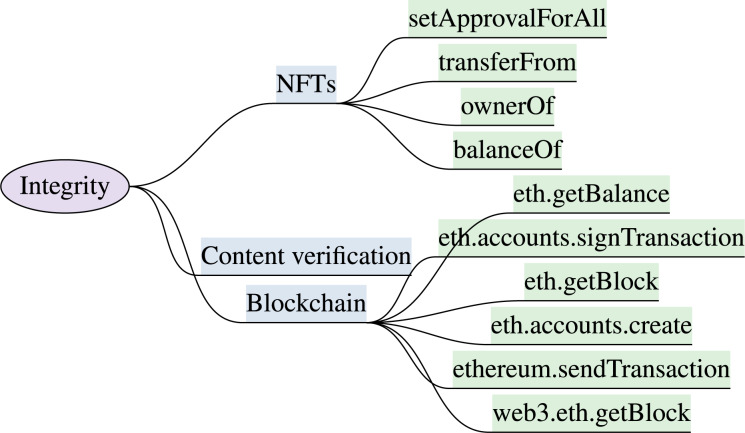
XR integrity model & implementation attributes (“Integrity”).

The virtual content, input, and output overlays must be precisely and accurately rendered. The virtual content and XR system configuration should be tamper-resistant and inaccessible to unauthorized users during an immersive session. The XR immersive experiences are vulnerable to various integrity attacks, including tracker attack, chaperone attack, overlay attack, and human joystick attack (Pearlman, 2020). A tracker attack manipulates the XR tracking system, a chaperone attack alters the security boundaries and safety zone, a human joystick attack gains control over users’ virtual controllers, and an overlay attack blocks users’ view in an immersive environment. The XR systems must verify the integrity of systems and associated libraries and dlls before usage. The overview of integrity features and corresponding development attributes are explained in this section and are shown in Fig. 4. The integrity features with security requirements and protection capabilities against immersive attacks are discussed in this section and outlined in Table 5.

**Table 5:** XR integrity features.

No.	Integrity features	Significance	Prevents
1	Age & Content verification	• Safe child interactions	• Inappropriate content & ads
		• Age-specific adverts	• Addiction
		• Time restriction to virtual exposure	• Virtual misconduct
			• Bullying & harassment
2	Immutable blockchain	• Content integrity	• Data reliability
		• Verifiable, Traceable, & Transparent transaction	• Tamper resistant
3	NFTs	• Evidence of virtual transaction	• Data tampering
		• Trust	• Double-spending attack
		• Ownership	• Sybil attacks
		• Authenticity of digital assets & virtual transactions	• Unauthorized access
			• Asset copying or duplication,
			• Fraudulent transactions
			• Ownership disputes

***Immutable blockchain:*** The immutable property of blockchain maintains a copy of the content in a block through the linked chain that ensures content integrity (Huynh-The et al., 2023). Moreover, blockchain technology incorporates asymmetric-key encryption and hash functions to ensure data integrity in the metaverse. Each activity or transaction is recorded as a block, and each block in a blockchain maintains a hash of the previous transaction (Luo et al., 2021). Since a single record or a block can not be altered without affecting the other linked blocks, by integrating the blockchain, metaverse users can ensure data reliability and are tamper-resistant (Zhang et al., 2021). The blockchain transaction and smart contracts in the metaverse are verifiable, tamper resistant, traceable, and transparent. The ownership of metaverse assets with the transaction details are stored on Ethereum blockchain. Maintaining blockchain records involves higher storage capacity, transaction times, and cost, as compared to regular transactions (Alrubei et al., 2020). Blockchain users pay high prices to secure their metaverse assets and ensure that the experience is safe and secure using Ethereum’s blockchain. Web3.js (Meta, 2022a), Ethers.js (Moore, 2023), Decentraland SDK (Decentraland, 2023a), EOSIO, Hyperledger Fabric (EOSIO, 2024; Hyperledger Foundation, 2024) are used to manage smart contracts, wallets, handle transactions and enable the trading of virtual assets. The relevant functions include *etc*.accounts.create, eth.getBlock, eth.accounts.signTransaction, ethereum.sendTransaction and eth.getBalance.

***NFTs:*** Blockchain assists in securing the metaverse transactions by providing evidence of the user’s activities (Weston, 2021). Metaverse provides a certificate of virtual assets purchases in the form of NFTs and provides an immutable guarantee of ownership (Nahar, 2022). NFT does not prevent the stealing of virtual assets, but it serves as evidence against the misuse of virtual assets. The crypto-currencies, NFTs, virtual currencies such as mana (Kriptomat, 2023) in decentraland ecosystem, and enjin coin (Enjin, 2023) in many metaverse platforms, are likely to become the metaverse value exchange (Sun et al., 2022). NFTs establish trust among stakeholders and ensure ownership and authenticity of digital assets and virtual transactions. NFT ensures the authenticity of transactions and prevents data tampering, double-spending, Sybil attacks, unauthorized access, illegal copying or duplication of virtual assets, fraudulent transactions, and ownership disputes (Chen et al., 2023; Huynh-The et al., 2023). Decentraland (Decentraland, 2023a) and Ethereum API (Ethereum, 2023) support NFT in the metaverse and XR systems. Functions including balanceOf to retrieve the number of NFTs owned by a specific address, ownerOf returns the current owner of a specific NFT, transferFrom to transfers ownership of an NFT, and setApprovalForAll sets or revokes approval for an operator, can be assembled to implement and manage NFTs.

***Age & Content verification:*** The security feature of age verification and virtual content integrity is introduced for safe child interaction and exposure in an immersive world to safeguard users from virtual misconduct, bullying, and harassment. Only age-specific adverts and virtual content must be permitted to avoid inappropriate content and ads in XR systems. The feature will protect and enforce time restrictions to avoid addiction and excessive use of VR platforms (Meta, 2024b; Media, 2024; Londono, 2024). APIs that support the implementation of this feature comprise Veriff, and Onfido (Veriff, 2023; Onfido, 2024a), with the functionality of document verification, age calculation, track transaction, and enforce policies. Facial tracking features could be established in XR systems to identify the user’s age. The ARCore (Google Developers, 2023), ARKit (Apple Inc, 2023), AgeChecker (AgeChecked Ltd, 2023), Jumio (Jumio Corporation, 2023), Onfido, and Veriff can be accommodated to render age restricted content.

### Interoperability

**Figure 5:**
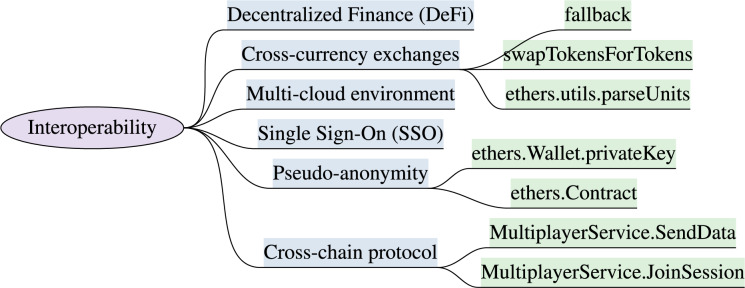
XR interoperability model & implementation attributes (“Interoperability”).

Interoperability is another major challenge to ensure a seamless transition between multiple sub-metaverse with immersive identities, virtual assets, and credentials. The metaverse and XR systems must be operable across integrated platforms, services, and devices. The identified interoperability features with high-level implementation attributes are discussed in this section. Figure 5 outlines the interoperability features and implementation attributes.

***Cross-chain protocol:*** The cross-chain protocol facilitates data exchanges between two or more blockchains within distinct virtual worlds. The seamless migration of users between these virtual worlds is facilitated by embedding cross-chain interoperability features (Huynh-The et al., 2023). Cross-chain allows the exchange of possessions and immersive belongings like avatars, NFTs, and virtual payments between XR environment. Unity multiplayer servicesSDK (Unity Technologies, 2025), is used for cross-platform interaction, and the functions comprised of MultiplayerService.JoinSession and MultiplayerService.SendData.

***Single Sign-On (SSO)*** is an authentication mechanism based on tokens, empowering the metaverse users to authenticate across diverse platforms and environments. It also supports interoperability by enabling virtual users to log in once and seamlessly access multiple metaverse and XR services. This streamlined login experience eliminates the necessity for distinct login credentials. SelfKey SDK (SelfKey Foundation, 2025), Auth0 (Auth0, 2024), Okta (Okta, 2024), Microsoft Azure AD B2C (Microsoft, 2024b), attributes support a single sign-on interoperability feature.

***Pseudo-anonymity:*** The feature of pseudo-anonymity is essential for XR users to partially hide the identities in virtual world (Nahar, 2022). Ethereum smart contracts are used to issue and verify virtual credentials to prove specific properties without revealing real identity. It supports interoperability between XR systems due to their shared blockchain-based underlying infrastructure and interconnected ecosystem. Ethereum wallets like Metamask, Trust Wallet, (Trust Wallet, 2023) *etc*. manages pseudonymous transactions. The libraries used to achieve these features include ethers.js, web3.js (Moore, 2023; Meta, 2022a) and corresponding functions are ethers.Contract, ethers.Wallet.privateKey, which facilitates the implementation of pseudonymous Ethereum accounts.

***Multi-cloud environment:*** In the metaverse, virtual assets created or purchased in one platform, can be used and sold on other platforms. *CISCO* is working on an open, integrated, multi-cloud solution to meet the metaverse business needs (TechCrunch, 2022). AWS SDK, Google Cloud (gcloud), and Azure SDK (Amazon Web Services Inc, 2024; Google, 2024a; Microsoft, 2024b) provide supportive functions to launch multi-cloud environment for XR systems.

***Cross-currency purchases & exchanges:*** The XR users can convert traditional currencies (fiat) into metaverse-native currencies, to make purchases within virtual worlds. Following are the relevant SDKs for currency exchanges, including Ethereum (Web3.js, ethers.js), Uniswap (Moore, 2023; Meta, 2022a) and their respective functions such as ethers.Wallet.sendTransaction, ethers.provider.getBalance, ethers.utils.parseUnits, ethers.Contract, ethers.utils.parseUnits, fallback, receive to handle incoming Ether transactions, and borrow, deposit, repay to borrow assets from lending protocols like Aave or Compound (OKX.COM, 2023), *etc*.

The decentralized finance (DeFi) (Investopedia, 2023) protocol can also be embedded for cross-currency transactions within the metaverse, and users can leverage DeFi services to swap between traditional and metaverse-native currencies. Smart contracts of decentralized exchange (DEX) protocols like Uniswap, SushiSwap (Tasty Software Solutions LLC, 2023), support cross-currency exchanges in the XR systems and to implement DEX’s smart contract. The XR systems needs to import the ABI (Application Binary Interface) (Alchemy, 2023) and respective methods such as swapExactTokensForTokens.

### XR forensics and incident reporting

The initial development of XR systems lacks forensic tools and processes to investigate virtual incidents. The INTERPOL (Interpol, 2022) and EUROPOL (De Bolle, 2022) have raised their concerns that virtual crimes and immersive attacks are being reported and the immersive XR systems lack efficient mechanisms to report, prevent, and address negative behaviors proactively. Moreover, the sensory gadgets and wearable devices worn on the the human body have minimal memory and human physiological indicators are hard to control. Metaverse forensics requires digital and virtual evidence such as avatars’ behaviors, virtual transactions, immersive interactions, and details of ownership of virtual assets. The analytical capabilities using blockchain transaction verification and collection of digital footprints from wearable devices such as head-mounted displays, haptic gloves, and VR goggles could provide evidence to some extent (Kempen, 2023). Multiple forensics artifacts are identified (Kim, Oh & Shon, 2023) to support forensics investigation in metaverse ecosystem such as the avatar’s profile name, teleport location, activity logs, and messages information. A major limitations of the multi-modal metaverse crime investigation is that the digital data, third-party servers, and cloud network complicate the authenticity of acquired data. The digital evidence can easily be modified, deleted, and obfuscated during the acquisition process for forensic analysis (Tiwari et al., 2025).

**Figure 6:**
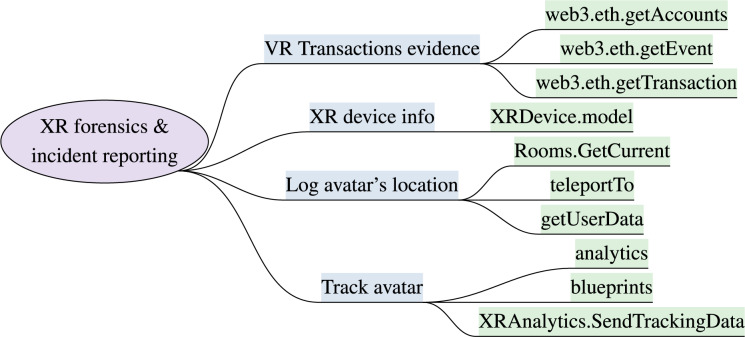
XR forensics model & implementation attributes (XR forensics and incident reporting).

The implementable security attributes to track, record, and report user’s behavior, interactions, and virtual incidents include XRAnalytics.SendTrackingData. The User Interface (UI) in unity3D (Unity Technologies, 2025) for incident reporting, capture blueprints feature in Unreal Engine (Epic Games Inc, 2024), and Google Analytics module to perform analysis on captured records. The decentraland SDK offers getUserData, teleportTo methods to log avatar’s location history. The XRDevice.model to gather details regarding make and model of device using Unity XR. VoIP.Start and Rooms.GetCurrent methods of Oculus sdk to monitor voice and users current sessions in VR rooms, respectively. web3.eth.getTransaction, web3.eth.getEvent, web3.eth.getAccounts methods are available to capture avatars transactions and activity using *web3.js* API. Figure 6 outlines the XR forensics features and implementation attributes.

Multi-jurisdictional issues in the metaverse amplify the complexity of immersive reality forensics. A cross-border internationally recognized legal framework needs to be established to facilitate metaverse forensics investigation (Guangjun et al., 2023). Laws and regulations in the digital environment tend to be reactive, imposing punishments after violations. However, immersive reality demands incentives for positive behavior. An immersive authority is required to investigate virtual incidents, perform forensics, and take further action, like removing and banning a user’s avatar from a particular metaverse on their reported incidents (Giannis & Nadhem, 2024). The security features of immersive surveillance and reporting are crucial to counter XR crimes. XR jail and virtual police is required to patrol the immersive environment (Ray, 2023b). The International Criminal Police Organization (ICPO), or international police (Interpol) (Interpol, 2022) has taken the initiative to set up a virtual space for the metaverse police and are investigating tools, techniques, and polishing skills to setup police in the Metaverse (Ray, 2023a).

## Statistical analysis and results

### XR security capability analysis

This section presents a statistical analysis to evaluate the capabilities and effectiveness of available SDKs and APIs in implementing the identified security features within the XR systems. The statistical analysis to assess the quality of development resources is illustrated in Tables 6, 7, 8 and 9 to achieve authenticity, privacy, integrity, and interoperability features, respectively. Table 6 shows that the privacy features of block user and safety bubble are present in most of the development libraries. We observe that the support of robust authentication attributes for the XR systems such as MetaSecure and Selfkey are available in very limited APIs as represented in Table 7. Our analysis shows that many critical security attributes are sparsely available in development resources. The complete builtin methods are only available for the fundamental security attributes such as support of block user, 3D pin, are currently available in most of the APIs. Table 10 assigns a statistical capability score to represent the capability and effectiveness of SDKs and APIs to achieve the security features in XR systems. The capability score is computed by assessing the presence of supportive attributes for each security feature, calculated as the ratio of supported attributes to the total number of identified features. Table 10 demonstrate that the Unreal Engine provides five supportive attributes among nine identified privacy features of XR (“Privacy”), and the assigned privacy score is 5/9 = 0.56. Similarly, the Unity XR achieves the highest privacy score of 7/9 = 0.78 and 6/13 = 0.46 to achieve authentication. The Ethereum API supports three attributes to achieve interoperability features among six with a score of 3/6 = 0.5. The summation of privacy, authenticity, integrity, and interoperability scores is computed to identify the most suitable APIs to implement immersive security features.

**Table 6:** Mapping of implementation attributes for XR privacy features in SDKs/APIs.

Feature	Decentraland API	High fidelity user API	VRChat API	Somnium space SDK	Oculus SDK	SteamVR unity plugin	Mixed reality toolkit	Unity XR SDK	Neos VR API	VR toolkit	UnrealEngine	Viveport SDK
Block user	✓	✓	✓	✓	✓	✗	✗	✗	✗	✗	✗	✗
Safety bubble	✗	✗	✗	✗	✓	✓	✓	✓	✗	✗	✓	✗
Fictional characters	✗	✗	✗	✗	✗	✗	✗	✓	✓	✗	✗	✗
Blurring background	✗	✗	✗	✗	✗	✗	✗	✓	✗	✗	✓	✗
End-to-end encryption	✓	✗	✗	✗	✓	✗	✗	✓	✗	✗	✓	✗
Teleportation	✗	✗	✗	✗	✗	✓	✓	✓	✗	✓	✗	✗
Avatar’s cloning	✗	✓	✓	✗	✗	✓	✓	✓	✗	✗	✓	✗
Private Space	✗	✓	✗	✗	✗	✗	✓	✓	✗	✗	✓	✓
Zero-knowledge proofs	✓	✓	✗	✗	✗	✗	✗	✗	✗	✗	✗	✗

**Table 7:** Mapping of implementation attributes for XR authenticity features in SDKs/APIs.

Feature	Unity XR SDK	Leap motion SDK	Oculus SDK	NeuroSky toolkit	OpenCV API	Vive SDK	Unreal Engine	Sensor fusion libs	BiometricPrompt API	Auth0 SDK	Microsoft azure MR	Google identity platform	OpenID connect	OAuth API	Decentraland SDK	Ethereum API	SelfKey SDK	ARCore API	WebAuthn API
3D pin	✓	✓	✓	✗	✗	✗	✗	✗	✗	✗	✗	✗	✗	✗	✗	✗	✗	✗	✗
Biometric	✗	✗	✗	✓	✓	✓	✗	✗	✗	✗	✗	✗	✗	✗	✗	✗	✗	✗	✗
Behavioral pattern	✓	✓	✗	✗	✗	✗	✓	✓	✗	✗	✗	✗	✗	✗	✗	✗	✗	✗	✗
Multimodel	✗	✗	✗	✗	✗	✗	✗	✗	✓	✓	✓	✓	✗	✗	✗	✗	✗	✗	✗
Blockchain	✓	✗	✗	✗	✗	✗	✗	✗	✗	✗	✗	✗	✗	✗	✓	✓	✗	✗	✗
Blockchain+ biometric	✓	✗	✗	✗	✗	✗	✓	✓	✗	✗	✗	✗	✗	✗	✗	✗	✗	✗	✗
Self key	✗	✗	✗	✗	✗	✗	✗	✗	✗	✗	✗	✗	✗	✗	✗	✓	✓	✗	✗
Continuous auth	✓	✗	✗	✗	✓	✗	✗	✗	✗	✗	✗	✗	✗	✗	✗	✗	✓	✓	✗
Proof of humanity	✓	✗	✓	✗	✗	✗	✓	✗	✗	✗	✓	✗	✗	✗	✗	✗	✗	✗	✗
Token-based	✗	✗	✓	✗	✗	✗	✗	✗	✗	✗	✗	✗	✓	✓	✗	✗	✓	✗	✗
PhotoCromic	✗	✗	✗	✗	✓	✗	✗	✗	✗	✗	✓	✗	✗	✗	✗	✗	✗	✗	✓
MetaSecure	✗	✗	✗	✗	✗	✗	✗	✗	✗	✗	✓	✓	✗	✗	✗	✗	✗	✗	✗
Zero trust	✗	✗	✗	✗	✗	✗	✗	✗	✗	✗	✓	✓	✓	✗	✗	✗	✗	✗	✗

**Table 8:** Mapping of implementation attributes for XR integrity features in SDKs/APIs.

Feature	Web3.js API	Ethereum API	Decentraland API	ARCore API	ARKit SDK
Immutable blockchain	✓	✓	✓	✗	✗
NFTs	✓	✓	✓	✗	✗
Age verification	✗	✗	✗	✓	✓

**Table 9:** Mapping of implementation attributes for XR interoperability features in SDKs/APIs.

Feature	Unity XR SDK	Ethereum API	Decentraland API	Uniswap API	Web3.js API	Google cloud	Microsoft mixed reality Toolkit	Auth0 SDK	SelfKey API
Cross-chain protocol	✓	✓	✓	✗	✗	✗	✗	✗	✗
Pseudo-anonymity	✓	✓	✓	✗	✓	✗	✗	✗	✗
Cross-currency exchanges	✗	✓	✗	✓	✓	✗	✗	✗	✗
Multi-cloud	✗	✗	✗	✗	✗	✓	✓	✗	✗
Decentralized Finance	✗	✗	✗	✓	✗	✗	✗	✗	✗
Single sign-on	✗	✗	✗	✗	✗	✗	✗	✓	✓

**Table 10:** SDKs/API with XR security attributes & functions.

SDKs/APIs	Privacy score (X1)	Authenticity score (X2)	Integrity score (X3)	Interoperability score (X4)	XR Defensive Capability score $\sum$ (X1, X2, X3, X4)
Decentraland API	$3/9 = 0.33$	$0/13 = 0$	$2/3 = 0.67$	$2/6 = 0.33$	$0.33 + 0 + 0.67 + 0.33 = 1.33$
High Fidelity API	0.44	0	0	0	0.44
VRChat SDK	0.22	0	0	0	0.22
Somnium Space API	0.11	0	0	0	0.11
Oculus SDK	0.33	0.23	0	0	0.56
SteamVR Plugin	0.33	0	0	0	0.33
Mixed reality Toolkit	0.44	0	0	0.17	0.61
Unity XR SDK	0.78	0.46	0	0.33	1.57
Neos VR API	0.11	0	0	0	0.11
Virtual reality Toolkit	0.11	0	0	0	0.11
Unreal Engine	0.56	0.15	0	0	0.71
Viveport SDK	0.11	0	0	0	0.11
Ethereum API	0	0	0.67	0.5	1.17
Uniswap API	0	0	0	0.33	0.33
Web3.js SDK	0	0	0.67	0.33	1
Google cloud	0	0	0	0.17	0.17
Auth0 SDK	0	0.15	0	0.17	0.32
SelfKey API	0	0	0	0.17	0.17
Leap motion SDK	0	0.15	0	0	0.15
NeuroSky Toolkit	0.08	0	0	0	0.08
OpenCV API	0	0.23	0	0	0.23
Vive SDK	0	0.08	0	0	0.08
Sensor Fusion API	0	0.08	0	0	0.08
BiometricPrompt API	0	0.08	0	0	0.08
Microsoft Azure mixed reality	0	0.38	0	0	0.38
Google identity platform	0	0.15	0	0	0.15
OpenID connect	0	0.08	0	0	0.08
OAuth API	0	0.08	0	0	0.08
ARCore API	0	0.08	0.33	0	0.41
ARKit SDK	0	0	0.33	0	0.33
WebAuthn API	0	0.08	0	0	0.08

### Security assessment of XR systems

In this section, a security analysis is conducted to assess the security strengths of prominent XR system and their resilience against immersive security concerns. The evaluation focuses on the identified XR security features implemented across leading metaverse and XR systems. The results are drawn from analyzing the acquired dataset from diverse sources, including XR product web pages, manuals, and the official app store. The compiled analysis reveals that only a few industries and technology giants, such as Microsoft, Facebook (Meta), and Decentraland (2023b), are allocating resources towards the implementation of security controls in the XR and metaverse systems, summarized in Tables 11 and 12. The Meta Facebook incorporates security features such as block user, fictional characters, personal boundary, and teleportation. The Meta’s Oculus Quest headset supports multi-model authentication and age verification. The Decentraland emphasizes token-based authentication, privacy features of teleportation, private space, support NFT, cross-currency, and pseudo-anonymity. The Microsoft HoloLens and Microsoft Mesh support personal boundary and teleportation feature of privacy. In addition, Microsoft Mesh offers a multi-cloud environment to ensure interoperability. The XR security assessment score is determined by evaluating each XR system based on the identified list of XR security features, including authenticity, privacy, integrity, interoperability and forensics capabilities. The score is calculated as a ratio of the embedded or supported security features to the total number of XR security features. The cumulative security score is obtained as the sum of assessment scores across all evaluated security features. The evaluation of security strength of prominent XR systems is presented in Table 13. The cumulative score of Decentraland and SomniumSpace is higher, which indicates that these two products have embedded most of the identified immersive reality security features.

**Table 11:** Leading XR systems with security features (Continued on next page).

*Authentication*		
**PIN/3D pattern**	**Multi-modal**	**Self key**
Supernatural (Meta Quest, 2023c)	Tobii Eye Tracker (Tobii, 2023)	Metamask (MetaMask, 2016)
VRChat (VRChat Inc, 2017)	Oculus Quest 2 (Oculus, 2020)	Blockcerts (Learning Machine, 2016)
STRIVR (STRIVR, 2015)	Manus Prime II (Manus, 2021)	CryptoKitties (Axiom Zen, 2017)
**Proof of humanity**	**Behavioral Bio-metric**	**Token-based**
Aftermath Islands (Aftermath Islands, 2021)	Unity MARS (Unity, 2023)	Decentraland (Decentraland, 2023b)
		Somnium space (Somnium Space, 2025)
		Sandbox (Abrol, 2023)
		Cryptovoxel (Crunchbase Inc, 2023)
**Photochromic**		
Magic Leap One (Magic Leap Inc, 2018)		
Vuzix Blade AR Glasses (Vuzix, 2018)		

*Privacy*		
**Block user**	**Blurring background & standerby**	**End-to-end encryption**
Meta Facebook (Meta Platforms Inc, 2021)	SoWork (SoWork Inc, 2021)	Somnium space (Somnium Space, 2025)
Microsoft Mesh (Microsoft, 2023)	Project Aria (Meta, 2023)	Mozilla Hubs (MozillaHubs, 2023)
VRChat (VRChat Inc, 2017)		Engage (ENGAGE, 2023)
STRIVR (STRIVR, 2015)		Decentraland (Decentraland, 2023b)
Snapchat Bitmoji (Snap Inc, 2024)		XR Wizards’ Mazer (Decentraland, 2023b)
**Teleportation**	**Private Space**	**Personal boundary & Safety bubble**
Rec Room (Rec Room, 2016)	Rec Room (Rec Room, 2016)	Meta Facebook (Meta Platforms Inc, 2021)
Decentraland (Decentraland, 2023b)	Decentraland (Decentraland, 2023b)	VRChat (VRChat Inc, 2017)
VRChat (VRChat Inc, 2017)	VRChat (VRChat Inc, 2017)	Rec Room (Rec Room, 2016)
Somnium space (Somnium Space, 2025)	Somnium space (Somnium Space, 2025)	Somnium space (Somnium Space, 2025)
Aftermath Islands (Aftermath Islands, 2021)		Microsoft HoloLens 2 (Microsoft, 2019)
Steam VR (Valve, 2023)		
Meta Facebook (Meta Platforms Inc, 2021)		
Roblox (Roblox Corporation, 2023)		
Microsoft HoloLens 2 (Microsoft, 2019)		
Spatial.io (Spatial Systems Inc, 2024)		
Microsoft Mesh (Microsoft, 2023)		
**Fictional characters**		
Meta Facebook (Meta Platforms Inc, 2021)		
VRChat (VRChat Inc, 2017)		
Rec Room (Rec Room, 2016)		
Snapchat Bitmoji (Snap Inc, 2024)		
Codec avatars (Wöbbeking, 2023)		

**Table 12:** Leading XR systems with security features.

*Integrity*		
**Age & content verification**	**Content integrity**	**Blockchain + NFT**
Oculus Quest 2 (Oculus, 2020)	Samsung Gear VR (SAMSUNG, 2023)	VIBEHub (VIBEHub.io, 2023)
Steam VR (Valve, 2023)	Meta 2 AR headset (The Verge, 2023)	Somnium Space (Somnium Space, 2025)
PlayStation VR		High Fidelity (High Fidelity Inc, 2023b)
		Decentraland (Decentraland, 2023b)

*Interoperability*		
**Blockchain & NFT**	**Cross chain protocol**	**Cross currency**
Leewayhertz (LeewayHertz, 2025)	Sandbox (Abrol, 2023)	Fortnite (Epic Games Inc, 2023)
Decentraland (Decentraland, 2023b)	CryptoMotors (CryptoMotors, 2023)	Decentraland (Decentraland, 2023b)
	Somnium Space (Somnium Space, 2025)	Somnium Space (Somnium Space, 2025)
		Cryptovoxel
		Sandbox (Abrol, 2023)
**Pseudoanonymity**		
Second Life (Linden Research Inc, 2023)		
Somnium Space (Somnium Space, 2025)		
Decentraland (Decentraland, 2023b)		
VRChat (VRChat Inc, 2017)		

*XR forensics*
**Crime reporting**
Fortnite (Epic Games Inc, 2023)
Rec Room (Rec Room, 2016)
Roblox (Roblox Corporation, 2023)
VRChat (VRChat Inc, 2025)

**Table 13:** Security features in XR systems.

XR systems	Authenticity score (Y1)	Privacy score (Y2)	Integrity score (Y3)	Interoperability score (Y4)	XR systems security assessment score $\sum$ (Y1, Y2, Y3, Y4)
Supernatural	1/13 = 0.07	0/9 = 0	0/3 = 0	0/6 = 0	0.07
VRChat	0.07	0.55	0	0.16	0.78
STRIVR	0.07	0.11	0	0	0.18
Tobii eye tracker	0.07	0	0	0	0.07
Oculus Quest 2	0.07	0	0.33	0	0.4
Manus Prime II	0.07	0	0	0	0.07
Metamask	0.07	0	0	0	0.07
Blockcerts	0.07	0	0	0	0.07
CryptoKitties	0.07	0	0	0	0.07
Magic leap one	0.07	0	0	0	0.07
Vuzix blade AR glasses	0.07	0	0	0	0.07
Aftermath Islands (1st)	0.07	0	0	0	0.07
Unity MARS	0.07	0	0	0	0.07
Decentraland	0.07	0.33	0.33	0.5	1.23
Somnium space	0.07	0.44	0.33	0.5	1.45
Sandbox	0.07	0	0	0.66	0.73
Cryptovoxel	0.07	0	0	0.16	0.23
Meta facebook	0	0.44	0	0	0.44
Microsoft Mesh	0	0.22	0	0	0.22
SoWork	0	0.11	0	0	0.11
Project Aria	0	0.11	0	0	0.11
Mozilla Hubs	0	0.11	0	0	0.11
Engage	0	0.11	0	0	0.11
XR Wizards’ Mazer	0	0.11	0	0	0.11
Rec Room	0	0.44	0	0.16	0.6
Codec Avatars	0	0.11	0	0	0.11
Aftermath Islands (2nd)	0	0.11	0	0	0.11
Steam VR	0	0.11	0.33	0	0.44
Roblox	0	0.11	0	0.16	0.27
Microsoft HoloLens	0	0.22	0	0	0.22
Fortnite	0	0	0	0.16	0.16

## XR Security standards, regulations and government strategies

The metaverse and its platforms evolve globally, so it is imperative to examine the associated data privacy and security laws. Metaverse and XR security policies, standards, and regulations must be established and agreed upon by stakeholders internationally, including XR users, investors, developers, legal experts, and human rights experts. An industry-wide code of conduct needs to be formulated to work together and to agree upon base principles. Currently, the metaverse lacks specific regulations, but certain security aspects fall under the available legal frameworks, encompassing data protection laws, intellectual property rights, and criminal statutes. In this section, global efforts and contributions are highlighted towards the formulation of security standards, guidelines, and frameworks for a virtual immersive ecosystem.

The ISO/IEC TR 23844:2023 document (ISO, 2023c) provides guidelines for using XR for educational purposes, and currently, ISOIEC JTC 1SC 24WG 11 (ISO, 2023a, 2023b) is working towards the standardization of XR technologies in the domain of health and safety. The XR Safety Initiative (XRSI) frameworks (XR Safety Initiative, 2025) have been developed which are intended to design evolving security and privacy laws for XR technologies. XRSI also incorporates existing privacy laws such as GDPR (European Union, 2018) and NIST (W3C Immersive Web Working and Community Groups, 2023). NIST (W3C Immersive Web Working and Community Groups, 2023) is also developing standards for immersive public safety situations. UL Standards & Engagement (ULSE) designed the ANSI/CAN/UL 8400 (ULSE Inc, 2023) standard for XR Technology to ensure the safety of electrical components involved in immersive applications. OpenXR and Open Metaverse Interoperability Group (OMG) (Object Management Group® Inc. (OMG®), 2023b) are contributing to ensure interoperability. The contributions of organizations working on XR and metaverse standards, including the XR security initiative (XRSI), Virtual World Society, Metaverse Standards Forum, and International Organization for Standardization (XR Safety Initiative, 2025; Virtual World Society, 2024; ISO, 2024) are outlined in Table 14.

**Table 14:** XR laws and standards.

XR security frameworks, laws & policies	
1. ISO/IEC JTC 1	The International Organization for Standardization (ISO) and the International Electrotechnical Commission (IEC) have developed standards for immersive technologies. The *ISO/IEC TR 23844:2023* document (ISO, 2023c) provides guidelines for the utilization of XR in learning, education, and training (LET). The *ISOIEC JTC 1SC 24WG 11* is working on the standardizing of XR health, safety, security, and usability aspects of technologies. It provides guidelines for an effective and user-friendly environment for various domains, including health and safety (ISO, 2023a, 2023b).
2. XR Safety Initiative (XRSI)	*XRSI* (XR Safety Initiative, 2025) leads the development of standards, and frameworks, including *Medical XR (Medical XR Advisory Council), Child Safety (Child Safety Initiative), Diversity and Inclusion (CyberXR Coalition), Trustworthy Journalism (Ready Hacker One), and the Metaverse Reality Check (The MRC)*. The designed *XRSI Privacy and Safety Framework* is a global collaborative effort, developed by experts from diverse fields to provide a baseline incorporating privacy requirements from GDPR (European Union, 2018), NIST (W3C Immersive Web Working and Community Groups, 2023) and other evolving laws. The *XRSI Privacy Framework* is derived from established NIST privacy framework and is aligned with the existing domestic and international legal and regulatory structures (XR Safety Initiative, 2025).
3. IEEE VR/AR Standards Group	IEEE VR/AR Standards Groups, advisory board, and the working group (IEEE Digital Reality, 2023; IEEE SA (Standards association), 2023) of experts define best practices, interoperability, and adaptability measures across multiple domains including gaming sector, health care, and safety measures.
4. WebXR API	WebXR API (Mozilla Foundation, 2023), webXR2 is a web standard that delivers AR/VR experience to users through the web browser without any specialized apps and supports Cross-Platform development and integration environment.
5. NIST XR Standards	The National Institute of Standards and Technology (NIST) has been involved in the research and development of standards for XR focusing on simulating immersive public-safety situations including human-robot interactions, fire environments, *etc*., and creating standards after performing usability testing (W3C Immersive Web Working and Community Groups, 2023).
6. ISO/IEC 27001	ISO/IEC 27001 is an internationally recognized standard that provides a framework for establishing, implementing, maintaining, and continually improving an information security management system. The certification indicates that the organization follows best practices in managing and securing information assets.
7. ANSI/CAN/UL 8400	*UL Standards & Engagement (ULSE)* designed the *ANSI/CAN/UL 8400* standard for XR Technology, which ensures the safety of XR electrical components used in immersive applications. This standard covers various devices, including head-mounted displays, holographic displays, smart glasses, and interactive simulators. It aims to address potential hazards such as visual issues, heat exposure, and biomechanical stress, complementing existing safety requirements (ULSE Inc, 2023).
8. AR for Enterprise Alliance (AREA)	As a global non-profit organization, AREA strengthens AR enterprises by identifying challenges and opportunities in the AR ecosystem and promoting constructive discussion among AR stakeholders (Object Management Group® Inc. (OMG®), 2023a).
9. Open Metaverse Interoperability Group (OMG)	The Open Metaverse Interoperability Group (OMG) is an open-source collaborative community of industrialists, experts, and researchers who are developing inter-operable technology in an open environment (Object Management Group® Inc. (OMG®), 2023b).
10. OpenXR	To ensure interoperability, OpenXR (Pavlik et al., 2023) an open standard for building VR and AR runtimes, provides a common API for different XR hardware.

Recently, many countries have taken initiatives toward developing XR policies and regulations to ensure the security and privacy of its stakeholders. These frameworks aim to address concerns such as users’ physical well-being, and data privacy, and help to create a secure and trusted Metaverse. The European Union (EU) is developing XR rules and policies to ensure industrial alliance and interoperability among public and private sector organizations (Bond, 2022). South Korea’s National Data Policy Committee is developing standards to ensure ethical behavior and to restrict bad actors in the metaverse (Pessarlay, 2022b). The Ministry of Industry and Information Technology in China is also creating and revising existing policies to safeguard virtual content. The BBFC (British Board of Film Classification) is working to safeguard kids immersive XR experiences. The globally established extended reality security standards, regulations, government efforts, data protection, and user safety laws are elaborated in Table 15. The adherence to the XR policies, established at the country or state level, is mandatory for XR communities, developers, and stakeholders in the specified regions. The researchers, governments and nations are actively involved in the planning, advancing, and formulating security and privacy laws specific to the XR environment, as detailed in Tables 14 and 15.

**Table 15:** XR governance and regulations.

Country/State governance & regulations for XR	
1. Europe	The European Union (EU) aimed to initiate global metaverse regulation and is developing rules and policies to ensure industrial alliance and interoperability among public and private metaverse firms (Bond, 2022). The EU’s regulations will introduce taxes on the metaverse network backbone, which hosts software to launch metaverse spaces (Hoppe, 2022). The *GDPR (General Data Protection Regulation)* (European Union, 2018) enforced within the European Union (EU) and European Economic Area (EEA) sets stringent rules that impact XR handling of personal records, user profiling, cross-border data sharing, *etc*. *European Commission’s Virtual Worlds Strategy* (European Commission, 2024) ensures an open, secure, trustworthy, technology shift towards virtual worlds and web 4.0 for EU’s citizens, businesses, and public institutions. The EU applies its existing security and privacy laws such as General Data Protection Regulation (GDPR), Digital Services Act (DSA), and Data Governance Act and Data Act to VR platforms (European Commission, 2024).
2. South Korea	Korea’s Ministry of Science and Information and Communication Technology (MSIT) has taken initiatives to mature the metaverse ecosystem and passed a *Virtual Convergence (Metaverse) Industry Promotion Act* to address the regulatory challenges of cross-industry technological convergence (Innovation Centre Denmark, 2024). South Korea *National Data Policy Committee* is raising the metaverse ethical concerns and is developing standards for the metaverse world for protection against bad actors and technology misuse (Pessarlay, 2022b). The South Korean framework for video gaming is incapable of handling the immersive challenges and showed concerns over minors subjected to metaverse harassment and sexual assault (Bond, 2022). The *VR content regulations* in *South Korea* aims to observe age rating and provide ethical standards to curb virtual harassment and offensive behavior in immersive reality projects.
3. Japan	A *Web3 policy office* has been established under the *Ministry of Economy, Trade and Industry (METI), Japan*. The office’s mandate is to strengthen policies for Web 3.0. business environment in Japan (Pessarlay, 2022a). According to the Japanese Ministry of Economy, *Web 3.0, blockchain, non-fungible tokens (NFTs), metaverse*, and digital market will boost Japan’s economy. The government is promoting efforts in this direction, *Japan’s VR and AR guidelines* (Bond, 2022; Pessarlay, 2022a) are designed for customer awareness and age rating to offer a safe virtual environment, especially for kids and disabled consumers. Japan has defined a strategy for the adoption of metaverse domain, and is building *Japan Metaverse Economic Zone* to deliver a secure, stable and open metaverse infrastructure known as *RYUGUKOKU (TBD)* (Virtual Dimension Center (VDC), 2025).
4. USA	A metaverse Strategy Team has been established under the *Holland & Knight* law firm for the metaverse projects that assist developers and stakeholders in following legal strategies for immersive life. Moreover, the metaverse strategy team guides online firms and entrepreneurs regarding evolving legal concerns and laws for virtual environments (Holland & Knight LLP, 2022). The *FTC ACT (Federal Trade Commission Act)* of the USA provides guidelines to ensure the consumer’s privacy and data protection practices in immersive reality, including AR and VR environments (Nguyen, 2023). NIST is actively participating in defining security and privacy standards and frameworks for the metaverse and XR (National Institute of Standards and Technology, 2022).
5. China	The Ministry of Industry and Information Technology in China is developing policies for virtual content. *Cybersecurity Law* in China addresses concerns regarding virtual data sharing between countries, and product assessment, and content censorship in XR (Wun & Tan, 2018). *Ministry of Industry and Information Technology (MIIT)* in china has establish a *Metaverse Standardization Group* to define metaverse regulations to promote healthy and orderly development (Shen, 2024; Li, 2023).
6. UK	The *BBFC (British Board of Film Classification)*, aids parents to safeguard kids by providing age classifications and content ratings for XR experiences (British Board of Film Classification, 2016).
7. CA	The *PIPEDA (Personal Information Protection and Electronic Documents Act)* (Intersoft Consulting, 2021) in Canada doesn’t explicitly deal with the XR security concerns but the fundamental principles of personal data collection, user tracking, and data breach reporting dealt with many security and privacy concerns of XR ecosystem and XR companies operating in Canada must adhere to these principles to ensure they are compliant with *PIPEDA*.
8. Australia	The *Australian Classification Board* plays a role in classifying and rating media, including films, video games, and virtual and augmented reality content (Stevens, 2023). The Australia’s independent regulator (*eSafety Commissioner*) (eSafety Commissioner, 2023) to ensure users online safety has identified and published a report on potential XR and the metaverse risks such as exposure to harmful content and the need for proactive safety measures in these evolving digital spaces.
9. Singapore	The *PDPA (Personal Data Protection Act)* (Tsaaro, 2023), regulates the disclosure of personal information, ensures user’s consent, and imposes penalties for manufacturers that violate its policies (Tsaaro, 2023; ICTLC Italy, 2023).
10. India	The working on *Data Protection Bill* of India enforces IoT, VR, and AR manufacturers to consume user’s personal information ethically (KPMG, 2022).
11. Brazil	The Brazilian *LGPD (General Data Protection Law)* ensures that VR vendors process only relevant data required for the VR experience, take users’ consent before data collection, protect against unauthorized disclosure, and cross-border data sharing (Law, 2022).
12. France	The French *CNIL (National Commission on Informatics and Liberties)* (Commission Nationale de l’Informatique et des Libertés (CNIL), 2023) enforces data privacy regulations of XR and takes users’ consent before processing their records, audits whether the XR applications are processing data by the law, and includes recommendations for data protection (Kalyvaki, 2023).
13. UAE	The UAE’s *Responsible Metaverse Self-Governance Framework* highlights the regulatory principles of metaverse privacy and ethical usage. The *Dubai International Financial Centre (DIFC)* and *DIFC Metaverse Platform*, is aligned with the *Dubai Metaverse Strategy* to define policies and attract innovators, talents and industrialist to build and enhance metaverse technology (Dubai International Financial Centre, 2023; UAE Minister of State for Artifical Intelligence, 2023; UAE Government, 2023). Moreover, the UAE’s cabinet resolution No. 111 of 2022 (United Arab Emirates Cabinet, 2022), establishes a comprehensive regulatory framework for virtual assets (VAs) and virtual asset service providers (VASPs) within the UAE. The resolution aims to enhance investors protection, compliance with anti-money laundering (AML) and counter-terrorism financing (CTF) laws to foster a secure environment for virtual transactions.

## XR Security recommendations

### XR domain intensive security prioritization

The XR technology is widely adopted in almost every domain of life. In this section, we have proposed security recommendations to prioritize the critical security features of a particular XR domain to meet the consumer’s requirements and safeguard the integrity of immersive reality systems.

- The security feature of content integrity is crucial for sensitive immersive domains such as health care, medicine, military, and educational training. These domains process highly sensitive data, which could have severe implications if compromised. Ensuring the content remains authentic, unaltered, and secure from unauthorized modification during immersive sessions is crucial to maintaining stakeholders’ trust in XR applications.
- Age and content verification with parental control are advised to be prioritized for kids’ safety in the immersive environment to avoid health hazards, XR addiction and negative interactions. The existing safety and precautionary frameworks, such as Oculus VR Safety and Warranty Information (Meta Quest, 2023b) should be implemented and enhanced for the virtual environment to ensure child safety and to offer real-time monitoring.
- Social and multi-contestant immersive applications are recommended to enable privacy features like personal boundaries, block virtual participants, and incident reporting, to avoid XR crimes like groping, cyberbullying, and immersive assault. Privacy features like avatars’ identical clones, private copies of the virtual world, and teleportation are essential for immersive social gatherings to avoid cyberstalking. The social XR applications can enforce penalties for misbehavior and must report and share incidents with virtual police like meta police.
- Virtual marketplaces are recommended to prioritize features of secure and authentic transactions with ownership of purchases using blockchain and NFTs.
- Educational immersive XR apps designed to conduct workshops, training, interviews, and exams are encouraged to identify and authenticate the end users during the entire session by launching a continuous authentication mechanism.
- User’s personal information and medical records must be processed and stored on users’ devices locally. The private data, inferred information, virtual appearance, and profile, including avatars’ voice, name, and physique, should remain hidden from the public, and XR applications must obtain users’ consent before sharing their data with a third party.
- The user-friendly identity verification procedures like proof of humanity and PhotoChromic identity management can be incorporated and prioritized for applications designed for emergency situations like military, disaster management, and medical consultations.

### XR security checklist

In this section, a security checklist is designed for the metaverse and XR systems. This checklist will enhance the security posture of the XR system and facilitate the evaluation of the system’s security strength. This will increase resilience to counter emerging XR security challenges. Following are some recommendations:

- To ascertain the security posture of a particular XR system, it is imperative to verify whether the underlying development environment is scheduled to receive timely updates and incorporate the latest security patches. This proactive measure mitigates the XR system from integrated platform vulnerabilities.
- To ensure system integrity, the XR applications should be downloaded exclusively from a trusted source, such as official app stores, to ensure that the applications are legitimate and free from malicious code.
- The XR systems should adhere to the available best practices from the initial stages of design and development. Compliance with the available guidelines, such as “The XR design guidelines” (Ultraleap, 2024) can be incorporated to develop a comfortable and user-friendly interface in the XR systems. “The guidelines for immersive virtual reality” (Michalak, 2017) by Intel can be embedded to emphasize users’ physical and digital safety in immersive land.
- The XR systems must clearly articulate policies governing data collection, usage, and storage, as well as the measures adopted to safeguard users’ physical and psychological health. These policies should be readily accessible and communicated to all stakeholders.
- The system should require explicit users’ consent for personal data collection, sharing, and access to integrated resources such as the camera, location, and microphone to ensure that the users are informed about the scope of data collection. The metaverse and XR applications should also acquire users’ consent about their virtual appearances and profiles, including avatars’ voices, names, physiques, *etc*.
- The system should support virtual content deletion, a fundamental requirement to avoid replication, breaches, and the misuse of users’ information.
- The XR system must confirm that the users’ virtually crafted content and assets are impenetrable and that copyright infringement policies are enabled.

## Limitations and future direction

The proposed work has some limitations, as it relies on the imported APIs and SDKs to extract implementable attributes to embed the security features in the XR systems. The outcomes of XR defensive model would substantially improve if the imported development libraries encompass extensive attributes and methods, to achieve the security objectives for the immersive environment. In the future, the number and quality of imported APIs and SDKs can be increased, along with the novel defensive approaches designed specifically for the immersive reality to enhance the security posture of the metaverse and XR systems. Collaborative efforts from XR stakeholders, researchers, cyber analysts, and government bodies are also increasing to ensure safety and security measures in XR platforms, holding individuals accountable in the event of immersive incidents. Governments and research communities emphasize developing strategies and frameworks, as procedural routines and continual processes to achieve security and privacy. Automated mechanisms need to be established to evaluate the security strength of a particular XR system. In the future, we plan to devise an automated XR security evaluation system and security metrics to gauge and scale the defensive strength of a targeted XR system.

## Conclusion

The evolving metaverse and rapid development of XR systems introduce security vulnerabilities and novel immersive threats to the physical, psychological, and digital well-being of its users. Proactive defense to counter novel immersive attacks and sophisticated virtual crimes is challenging in the integrated XR and metaverse ecosystem. In this research, we offered a comprehensive XR defensive model to determine the defensive features with security strengths and mitigation capabilities to reduce the threat landscape of immersive crimes and security challenges. The proposed defensive model and implementation attributes will provide a defensive baseline for XR stakeholders, researchers, developers, and policymakers to counter potential XR attacks and sophisticated immersive crimes. The global effort, security standards, and frameworks are outlined in this article to incorporate the defensive measures proactively. The proposed XR security recommendations will further enhance the security posture of XR systems and reduce the threat landscape of immersive crimes and security challenges.

## Supplemental Information

10.7717/peerj-cs.3054/supp-1 Supplemental Information 1 Acronyms.

## Additional Information and Declarations

### Data Availability

The following information was supplied regarding data availability:

The data are available at GitHub:

- https://github.com/qamarsara/XR-Security-Development-Resources.

- qamarsara. (2025). qamarsara/XR-Security-Development-Resources: XR-Security-Development-Resources (v1.2.0). Zenodo. https://doi.org/10.5281/zenodo.15817582
