# Supplementary material for: A defensive model and implementation baseline for the metaverse and extended reality systems
Source: PeerJ Comput Sci. 2025 Aug 29;11:e3054. doi: 10.7717/peerj-cs.3054 (PMC12453801; doi:10.7717/peerj-cs.3054)
Supplement: Supplemental Information 1 [file peerj-cs-11-3054-s001.pdf]

Table 1: XR and Metaverse Security Acronyms

**Acronym**	**Definition / Description**
XR	Extended Reality – Umbrella term covering AR, VR, and MR
AR	Augmented Reality – Enhances the real world with digital overlays
VR	Virtual Reality – Fully immersive digital environment replacing real-world input
MR	Mixed Reality – Blends physical and digital worlds where both coexist and interact
Metaverse	A persistent, shared digital space integrating XR technologies and social presence
MITM	Man-in-the-Middle – Interception attack where adversary relays or alters communication
Object Erasure	Attack removing or hiding digital objects via occlusion or rendering manipulation
Virtual Spoofing	Deceptive placement of objects or avatars to manipulate perception
XR Hijacking	Unauthorized control or redirection of XR sessions or spatial context
Environment Poisoning	Inserting malicious elements into a shared XR environment
Render-based Attacks	Exploiting rendering protocols to obscure, manipulate, or disable digital elements
Authentication Drift	Decline in biometric or behavioral match accuracy over time
Continuous Authentication	Persistent user validation through behavioral or physiological signals
Zero Trust	Security model assuming no implicit trust, enforcing strict identity validation
Privacy Sandbox	Controlled data access framework to preserve user privacy in immersive environments
OpenXR	Cross-platform API by Khronos Group for XR applications and devices
ARCore	Google SDK for building AR experiences on Android
ARKit	Apple SDK for AR development on iOS
Vuforia	SDK for AR development with robust image recognition
Unity XR	Unity's XR framework supporting AR/VR/MR development
Unreal XR	XR development environment in Unreal Engine with support for immersive platforms
Guardian System	Safety boundary system used in VR (e.g., Oculus) to prevent real-world collisions
Reality Privilege Escalation	Gaining unauthorized control over spatial or contextual XR layers
Identity Spoofing	Mimicking another user/avatar in virtual environments
Sensor Tampering	Attacks targeting device input like IMUs, GPS, or cameras

Acronym	Definition / Description
MFA	Multi-Factor Authentication – Requires multiple credentials for authentication
Azure Cognitive Services	AI services and APIs by Microsoft Azure
Azure Mixed Reality Services	XR development tools and SDKs from Azure
Auth0	Identity platform for authentication and authorization
BehavioSec	Behavioral biometrics SDK/API for fraud detection
TypingDNA	Keystroke dynamics authentication API
UnifyID	Passive authentication using behavioral signals
BioCatch	Behavioral biometrics platform for identity fraud prevention
FaceTec	3D face authentication and liveness detection SDK
Onfido	Identity verification using biometric and document checks
iProov	Biometric face verification with liveness assurance
uPort	Self-sovereign identity system based on blockchain
Web3.js	Ethereum JavaScript API for blockchain interaction
Azure Blockchain Service	Microsoft service for managing blockchain networks
Hyperledger Fabric	Permissioned blockchain infrastructure
Solana	High-performance blockchain platform
EOSIO	Blockchain platform for scalable dApps
Okta	Identity and access management service
Microsoft Entra ID	Microsoft's rebranded Azure Active Directory for identity management
Google Cloud Identity	Google's IAM solution
Azure AD B2C	Customer identity and access management platform
Amazon Web Services	Cloud services provider offering SDKs for identity, ML, and more
Veriff	Identity verification platform for real-time biometric authentication
ISO	International Organization for Standardization, Developer of global standards (incl. security and XR)
Snapchat	Social media platform with AR/XR content delivery and lenses
Mixed Reality Toolkit (MRTK)	Microsoft toolkit for MR development
Snap AR SDK	Snap's SDK for augmented content creation
Unity XR SDK	Cross-platform XR development environment
Unreal Engine XR	XR content development platform by Epic Games
Oculus SDK	SDK for developing apps for Oculus VR headsets
WebXR API	Browser API for immersive XR experiences
Azure Remote Rendering	Microsoft service for rendering XR content securely

**Acronym**	**Definition / Description**
AWS Sumerian	Amazon tool for XR environment creation
Zero Trust API	APIs enforcing continuous identity validation
COPPA	Children's Online Privacy Protection Act
GDPR	General Data Protection Regulation
Netverify (Jumio)	Document-based identity and age verification SDK
Onfido	Biometric and document verification SDK
Yoti	Blockchain-based ID and age verification platform
Azure Content Moderator	API for text, image, and video moderation
Google Perspective API	Toxicity and content moderation API for chat/text
Unity Ads SDK	Advertisement management with age-based targeting
Meta Quest SDK	For XR app development and user authentication
AWS Cognito	Identity federation and SSO integration
OAuth 2.0 / OpenID Connect	For SSO and user identity verification
TrustArc / OneTrust	Compliance SDKs for GDPR, COPPA, etc.
ZKP	Zero-Knowledge Proof – Cryptographic method to prove possession of data without revealing the data itself
zk-SNARK	Zero-Knowledge Succinct Non-Interactive Argument of Knowledge – Efficient type of ZKP used for privacy-preserving authentication
SSI	Self-Sovereign Identity – A decentralized identity framework where users control their digital identities
AIGC	AI-Generated Content – Content generated by AI models, often in immersive or creative environments
JWT	JSON Web Token – A compact token format used in token-based authentication for secure transmission
Token-Based Auth	Authentication scheme using secure tokens (hardware/software) instead of direct credentials
3D Pattern Auth	Authentication based on 3D gesture or pattern input within XR interfaces
Multi-Modal Auth	Use of multiple authentication factors (e.g., biometrics + passwords) for improved security
Biometric Behavior	Unique user traits like gaze, gesture, voice, or typing patterns used for identification
Blockchain ID	Identity system based on decentralized blockchain ledger and cryptographic credentials
MetaMask API	API provided by MetaMask wallet for blockchain-based identity and transaction management
Web3.js	JavaScript library for interacting with Ethereum-based blockchains

Acronym	Definition / Description
OpenXR	Open standard API for accessing XR hardware and platforms in a unified way
Chameleon Signature	A trapdoor hash-based signature that provides non-repudiation and unlinkability without ZKP
BlockMaze	A zk-SNARKs-based blockchain model designed for privacy of XR transactions
Veramo	A JavaScript framework for building decentralized identity apps and APIs
uPort	Identity system enabling users to register and control digital identity on Ethereum
Avatar Hijacking	Attacker gains control of user's digital avatar in immersive environments
Semantic Injection	Malicious tampering of semantic or contextual XR data, e.g., in AI-generated content
Credential Stuffing	Automated attack where stolen credentials are used to gain unauthorized access
Digital Asset Theft	Unauthorized access or claim to virtual items like NFTs or crypto-tokens
Session Hijacking	Taking over a valid user session to impersonate and exploit access
SSI	Self-Sovereign Identity: user-owned decentralized digital identity.
SSO	Single Sign-On: authenticate once to access multiple systems.
IAM	Identity and Access Management: controls who accesses XR/VR systems.
AI Moderation API	APIs using AI to detect harassment, abuse, and objectionable content in XR.
OAuth	Open-standard for access delegation (authorization) in APIs.
PoI	Proof of Identification – a consensus algorithm in blockchain-based identity systems
NFT	Non-Fungible Token – digital asset used for ownership proof in metaverse
SSIM	Structural Similarity Index – used in content protection and tampering detection
ZTA	Zero Trust Architecture – cybersecurity model enforcing continuous authentication
JWT	JSON Web Token – a compact token used for secure data transmission
OAuth 2.0	Authorization framework for delegated access
OpenID Connect	Identity layer on top of OAuth 2.0 for user authentication
SelfKey	Decentralized identity management platform using blockchain and POI
PhotoCromic	Identity authentication using biometric + image + blockchain data

**Acronym**	**Definition / Description**
Auth0	Identity management platform supporting SSO and MFA in immersive apps
Firebase Auth	Google-backed authentication SDK for XR and web apps
Keycloak	Open-source identity and access management supporting SSO/MFA
Okta	Commercial identity-as-a-service platform for SSO and user identity
MSAL	Microsoft Authentication Library for Azure AD-based authentication
Blockchain	Immutable ledger used for identity, asset ownership, and data integrity
Facial Recognition	Biometric authentication using facial data
Behavioral Biometrics	Authentication using user behavior like gestures, typing patterns
Continuous Authentication	Ongoing identity verification throughout a session
Device Attestation	Verifying the trustworthiness of devices accessing XR/Metaverse
Bystander Privacy	Protection against unintended exposure of people near XR users
Sybil Attack	Attack using multiple fake identities – mitigated by SelfKey and PoI
Phishing	Deceptive login attempts – prevented via token-based and MFA methods
Replay Attack	Reuse of credentials or packets – mitigated by session tokens
Impersonation	Pretending to be another user – mitigated by biometric/blockchain IDs
Chaperone Attack	Attack that disables or modifies safety boundaries in VR environments
Human Joystick Attack	Attack where adversaries control user movement within a virtual environment
Overlay Attack	Attack that disrupts the user's vision by injecting malicious virtual content
Zero Trust	A security model assuming no implicit trust; enforces continuous verification
MFA	Multi-Factor Authentication – uses multiple credentials for authentication
IAM	Identity and Access Management – governs access to digital resources based on identity
Okta SDK	Identity platform SDK with built-in MFA, session control, and user management
Auth0 SDK	Identity service SDK supporting OAuth2/OIDC, adaptive MFA, and access control
Azure AD SDK	Microsoft identity SDK with Conditional Access and OAuth2-based login

**Acronym**	**Definition / Description**
Google Cloud Identity SDK	Google identity SDK with ID token auth, phone-based MFA, and SSO support
OAuth2	Open standard for delegated authorization using access tokens
OIDC	OpenID Connect – an identity layer on top of OAuth2 for authentication
JWT	JSON Web Token – a compact, URL-safe token format used in identity assertions
